# Assessment and Application of Acylcarnitines Summations as Auxiliary Quantization Indicator for Primary Carnitine Deficiency

**DOI:** 10.3390/ijns11020047

**Published:** 2025-06-19

**Authors:** Haijuan Zhi, Siyu Chang, Ting Chen, Lili Liang, Wenjuan Qiu, Huiwen Zhang, Xuefan Gu, Lianshu Han

**Affiliations:** Department of Pediatric Endocrinology and Genetic Metabolism, Xinhua Hospital, Shanghai Institute for Pediatric Research, Shanghai Jiaotong University School of Medicine, Shanghai 200092, China; haijuan_zhi@shsmu.edu.cn (H.Z.); changsiyu2148@xinhuamed.com.cn (S.C.); chenting02@xinhuamed.com.cn (T.C.); lianglili@xinhuamed.com.cn (L.L.); qiuwenjuan@xinhuamed.com.cn (W.Q.); zhanghuiwen@xinhuamed.com.cn (H.Z.); guxuefan@xinhuamed.com.cn (X.G.)

**Keywords:** primary carnitine deficiency, free carnitine, acylcarnitine summation, machine learning, screening and diagnosis, false positive, rare disease

## Abstract

Background: Newborns are referred primary carnitine deficiency (PCD) when a low free carnitine (C0) concentration (<10 μmol/L) is detected, leading to high false-positive referrals. To improve the follow-up protocol for PCD, various acylcarnitines and the summations were comprehensively evaluated in the present study. Methods: A retrospective study was performed using samples due to low C0 concentration. Data were available for 72 patients with genetically confirmed PCD, whereafter C0 with the selected sum of (butyrylcarnitine (C4) + isovalerylcarnitine (C5)) was validated in an additional cohort study including about 80,000 samples. Results: In the discovery study, C4, acetylcarnitine (C2) and C5 exhibited significant discriminant power in distinguishing PCDs from NoPCDs. The area under the ROC curve (AUC) was 99.792% (C4), 98.715% (C2) and 98.620% (C5). The excellent performances in sensitivity, specificity, negative predictive value, positive predictive value (PPV) and accuracy indexes suggested that C4, C2 and C5 would be ideal auxiliary indicators in improving the diagnostic performance of C0 for PCD. Multivariate ROC curve-based exploratory analysis showed that C5, C4 and C2 were the most top-ranked features in differentiating PCDs from NoPCDs. AUC for C4 + C5 was the highest with a cutoff required for 100% sensitivity at 0.181 μmol/L. In the validation cohort, adding C4 + C5 in the NBS program could elevate PPV from 0.75% to 1.54%. Conclusions: Our work revealed that C4 + C5 summation should be used as the auxiliary quantization indicator to reduce false-positive results for PCD.

## 1. Introduction

Primary carnitine deficiency (PCD, MIM 212140) is an autosomal recessive disorder caused by mutations in the *SLC22A5* gene, encoding the Organic Cation Transporter Novel 2 (OCTN2) protein [1,2,3,4]. OCTN2 can maintain intracellular free carnitine (C0) concentrations at normal levels by transporting carnitine into cells [5,6]. Carnitine is essential for transferring long-chain fatty acids from cytosol to mitochondria for subsequent beta oxidation [2,7,8]. Most PCD reported cases occur in infants and very young children. These patients often display an acute metabolic presentation with symptoms, such as hypoketotic hypoglycemia, elevated ammonia levels in blood, organic acidosis, and sudden death [2,7,9]. Onset is extremely rare in adulthood, and the clinical manifestations include progressive cardiomyopathy, myopathy, and encephalopathy due to hypoketotic hypoglycemia and hyperammonemia. The diagnosis and specific treatment of these age groups may be delayed for years [10,11]. The incidence of PCD varies in different countries, depending on the studied population, ranging from 1:300 in the Faroe Islands to 1:20,000–142,000 in Japan, Australia, or the USA [7,10,12]. Many large-scale Newborn Screening (NBS) studies have shown that PCD was the most prevalent fatty acid oxidation disorder in China, the incidence varying from 1:9000 to 1:34,000 [12,13,14,15,16,17].

Early diagnosis and timely supplementation of L-carnitine in the neonatal period are essential to achieve optimal clinical outcomes [8,18]. PCD is diagnosed based on levels of C0 and acylcarnitines, genetic mutation and clinical symptoms [10,12,19,20]. C0 and acylcarnitines are present at extremely decreased levels in PCD patients. However, transplacental transport of carnitine from mothers during pregnancy may lead to a low C0 level in neonates [12,21,22,23]. It is noteworthy that prematurity, malabsorption, malnutrition, and several inherited errors of metabolism, including organic acidemias and fatty acid oxidation defects, often lead to secondary carnitine deficiency [5,12,18,22]. These conditions represent pitfalls for the diagnosis and management of PCD neonates. Additionally, the currently used MS/MS-based markers for PCD diagnosis have undesirable performances, leading to high rates of false positives and occasional false negatives [13,16]. Follow-up of false positives is challenging, requiring a long period of confirmatory testing, including carnitine transporter activity measurement in cultured fibroblasts and/or *SLC22A5* gene sequencing [15,23]. The prolonged uncertainty concerning the health of newborns during this extensive assessment can induce significant anxiety in the families regarded. A faster exclusion of PCD diagnosis may reduce harm from false-positive referrals. Consequently, several algorithms for PCD screening have been proposed, including different thresholds for C0 and other biomarkers, molecular sequencing of *SLC22A5* and functional confirmation by carnitine uptake assay on skin fibroblasts [24].

At present, only a few countries apply secondary biomarkers in order to decrease false-positive rates for PCD [10,24]. Utilizing the sum of propionylcarnitine (C3) and palmitoylcarnitine (C16) on dried blood spot (DBS) < 2 µmol/L, in addition to a cutoff value of C0 < 10 µmol/L, allowed for a significant reduction in scree-positive cases and did not increase false-negative cases [25,26]. The positive predictive value (PPV%) increased from 7.1 to 13.0 [25]. Whilst acylcarnitines and their summations may be promising markers for the early differentiation of healthy newborns from PCD-affected newborns, information on acylcarnitines and their summations is limited. Furthermore, the optimization of newborn screening indicators and improvement in PPV are major challenges faced by newborn screening. According to the “Consensus on Primary carnitine deficiency Screening, Diagnosis and Treatment” in China, neonates with C0 < 10 µmol/L or C0 of 10~15 µmol/L, accompanied by a variety of acylcarnitine reductions, need to be recalled for review [27]. However, how to quantify multiple acylcarnitine levels is rarely reported in the literature.

In the present study, performances of free carnitine, specific acylcarnitines and their summations were comprehensively investigated on a larger scale to identify more effective NBS markers for PCD deficiency. This study demonstrates that the sum of butyrylcarnitine (C4) and isovalerylcarnitine (C5) could be used as an auxiliary diagnostic indicator in early discriminative tests for PCD.

## 2. Material and Methods

### 2.1. Research Subjects

The main objective of this study was to investigate the optimal auxiliary diagnostic indicators and significant changes in the acylcarnitines profiles of patients with PCD. A total of 72 PCD patients were recruited from 2003 to 2024. Detailed results of genomic confirmatory tests are listed in Supplementary Table S1. Infants with normal C0 levels and no clinical symptoms suggestive of metabolic disease were enrolled as the NonPCD group (*n* = 80). The utilities of C0 and various acylcarnitines and their summations in DBSs as markers for PCD were evaluated by comparing the data of the two groups.

To further validate the practicality of the selected auxiliary diagnostic indicators, an additional NBS cohort including 85,404 newborns was collected. Among them, 400 infants primarily displayed low C0 levels. Among them, 376 newborns were successfully recalled. Eventually, 3 newborns were diagnosed with PCD, yielding a PPV of 0.75% (3/400).

### 2.2. NBS Test for PCD

DBSs were collected from the heel pricks 72–120 h after birth. DBS samples were sent by cold chain transportation after natural air drying to Newborn Screening Center for tandem mass analysis. Both derivatized and underivatized methods were used for analyzing levels of amino acids, free carnitine, and acylcarnitines (API4000/API4500, Applied Biosystems, Foster City, CA, USA) [17,28]. The C0 cutoff value in our Newborn Screening Center was 10–60 µmol/L. Newborns in the initial screening with positive results (C0 < 10 µmol/L) were recalled and reexamined. The suspected PCDs were referred for confirmatory *SLC22A5* gene mutation analysis.

### 2.3. Confirmatory Tests for PCD

Genetic analysis for PCDs was conducted according to the methods described in previous research with minor modifications after receiving informed consent [4,17]. Genomic DNA was extracted from peripheral blood leukocytes. All exons and flanking intron regions containing *SLC22A5* were amplified using a polymerase chain reaction and analyzed on an automated DNA sequencer (Applied Biosystems, ABI3700). The *SLC22A5* gene mutations were analyzed using Sanger sequencing. The mutations were identified using a normal human *SLC22A5* sequence as a reference (NM_003060.4). Patients were diagnosed with PCD based on C0 levels, genetic mutations, and clinical symptoms. Newborns with two variants in the *SLC22A5* gene were diagnosed with PCD. The suspected positives carrying one variant or not, performing genetic testing but with persistently lower C0 values until therapy, were diagnosed with PCD [17].

### 2.4. Statistics

The sample distribution was determined using the Kolmogorov–Smirnov normality test. For statistical comparisons, Student’s *t*-test or Mann–Whitney U-test followed by the post hoc tests (Dunnett’s test) was utilized for the normal or non-normal distributed data, respectively.

Receiver operating characteristic (ROC) curves were generated to evaluate the sensitivity and specificity of free carnitine and various acylcarnitines and their summations as markers for PCD. Univariate and multivariate ROC curve analyses were performed in MetaboAnalyst 6.0. Multivariate ROC curves were performed based on three multivariate algorithms: support vector machines (SVMs), partial least squares discriminant analysis (PLS-DA), and random forests (RFs). ROC curves were generated by Monte-Carlo cross-validation (MCCV) using balanced sub-sampling. In each MCCV, two-thirds of the samples (2/3) were used to evaluate the feature importance. The most important features were then used to build classification models. The established models were then validated on the samples that were left out (1/3). The procedure was conducted multiple times to generate the confidence interval and performance of each model.

The area under the curve (AUC), fold changes (FC), *p*-values and false-discovery rate (FDR) were used to evaluate the significance of the respective acylcarnitines as potential diagnostic biomarkers. All the *p*-values were corrected by the false-discovery rate using the Benjamin–Hochberg method. The adjusted *p*-value (*q*-value) of 0.05 was taken as the significance level.

## 3. Results

### 3.1. Diagnostic Performances of Currently Used Free Carnitine and Acylcarnitines for PCD

Supplementary Table S2 shows levels of C0 and individual acylcarnitine [acetylcarnitine (C2), C3, C4, C5, caproylcarnitine (C6), caprylylcarnitine (C8), actinylcarnitine (C10), lauroylcarnitine (C12), myristoylcarnitine (C14), C16, octadecylcarnitine (C18)] in DBSs in the present study. In the PCD (*n* = 72) versus the NoPCD (*n* = 80) population, the C0 median concentration [interquartile range (IQR)] was 5.839 [4.392–7.201] and 26.418 [21.973–32.944] μmol/L, respectively (Supplementary Table S2).

The discriminant performances of C0 and individual acylcarnitines were investigated using ROC analysis (Table 1). AUC with the optimal cutoff was highest for C0 (100.000%), followed by C4 (99.792%), C2 (98.715%) and C5 (98.620%). This analysis evidenced that the currently used C0 is an optimal primary marker for PCD. The sensitivity, specificity, negative predictive value (NPV), PPV and accuracy indexes in Table 1 suggested that C4, C2 and C5 might be ideal auxiliary indicators in improving diagnostic performance of C0 for PCD.

**Figure 1:**
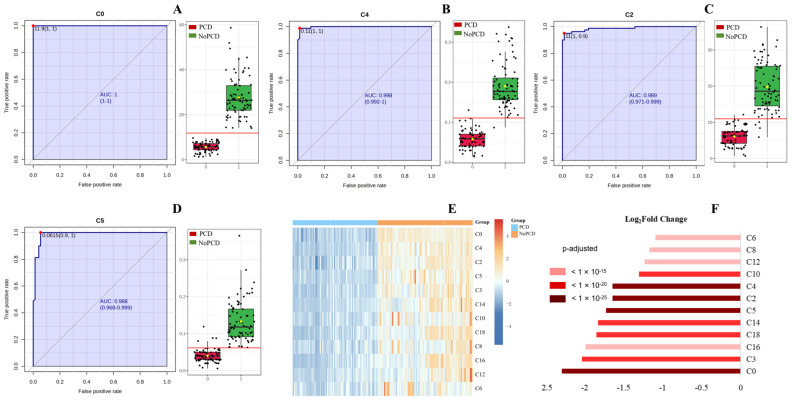
ROC curves and corresponding box plots of (**A**) C0, (**B**) C4, (**C**) C2, and (**D**) C5. The notch indicates the 95% confidence interval around the median of each group. The optimal cutoff is indicated with a horizontal red line on the boxplot. The mean concentration of each group is indicated with a yellow diamond. Each dots represents one individual sample. (**E**) Cluster heatmap and (**F**) fold changes of free carnitine and various acylcarnitines in dried blood specimens from PCDs and NoPCDs. PCD, primary carnitine deficiency; ROC, receiver operator characteristic; AUC, area under the curve; C0, carnitine; C2, acetylcarnitine; C3, propionylcarnitine; C4, butyrylcarnitine; C5, isovalerylcarnitine; C6, caproylcarnitine; C8, caprylylcarnitine; C10, actinylcarnitine, C12, lauroylcarnitine; C14, myristoylcarnitine; C16, palmitoylcarnitine; C18, octadecylcarnitine.

Figure 1 shows the ROC curves and corresponding box plots of C0, C4, C2 and C5 in DBSs in the PCDs and NoPCDs. All of these markers could clearly distinguish PCDs from NoPCDs. Supplementary Figure S1 presents ROC curves of the other specific acylcarnitines. Cluster heatmaps and fold changes of free carnitine and specific acylcarnitines (Figure 1E,F) show that C0, C4, C2 and C5 had the most significant changes (*q* value < 10^−25^). C3 and C16 displayed lower levels in patients, with the *q* value < 10^−20^ and < 10^−15^, respectively.

### 3.2. Machine Learning-Assisted Identification of Candidate Acylcarnitines for PCD Diagnosis

**Figure 2:**
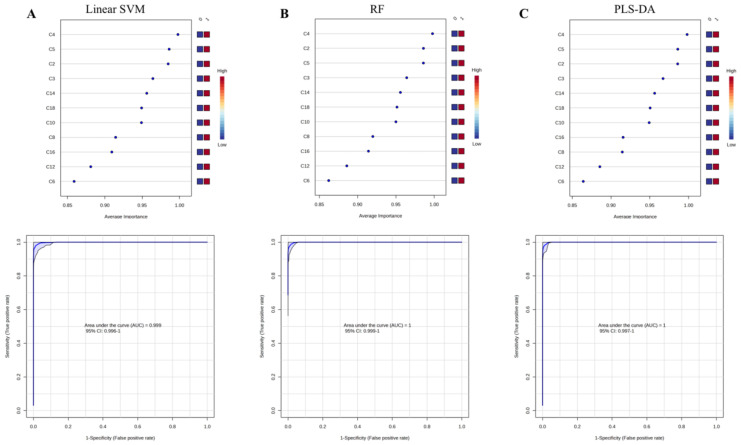
Average important features obtained from multivariate ROC curve-based exploratory analysis and the corresponding ROC curves of C5, C4 and C2. (**A**) linear surport vector machine-Linear SVM. (**B**) random forest-RF. (**C**) partial least square-discriminant analysis-PLS-DA. AUC, area under the curve; C2, acetylcarnitine; C3, propionylcarnitine; C4, butyrylcarnitine; C5, isovalerylcarnitine; C6, caproylcarnitine; C8, caprylylcarnitine; C10, actinylcarnitine, C12, lauroylcarnitine; C14, myristoylcarnitine; C16, palmitoylcarnitine; C18, octadecylcarnitine.

Multivariate ROC curve-based exploratory analysis was performed for identifying the optimal auxiliary diagnostic markers for PCD. ROC curve analyses were performed based on three multivariate algorithms: SVM, PLS-DA, and RF. Figure 2 shows the average important features obtained from multivariate ROC curve-based exploratory analysis. C5, C4 and C2 were identified as the most top-ranked features in all three methods. The AUC for the diagnosis of PCD in the studied cohort ranged from 0.999 to 1, with confidence intervals (CI) of 0.996–1, 0.997–1 and 0.999–1. To mine features having the most optimal performance and suitability for PCD diagnosis, summations of the top five features (C4, C5, C2, C14, C3) were selected and evaluated in discriminating PCDs from NoPCDs.

### 3.3. Diagnostic Accuracy Evaluation of Acylcarnitines Summations for PCD

Supplementary Figure S2 shows box plots of acylcarnitines summations in DBSs. Supplementary Table S3 shows individual acylcarnitines summation levels (C4 + C5, C4 + C2, C4 + C14, C4 + C3, C5 + C2, C5 + C14, C5 + C3, C2 + C14, C2 + C3, C14 + C3, C4 + C5 + C2, C4 + C5 + C14, C4 + C5 + C3, C5 + C2 + C14, C5 + C2 + C3, C2 + C14 + C3, C4 + C5 + C2 + C14, C5 + C2 + C14 + C3, C4 + C5 + C2 + C14 + C3, C3 + C16) in DBSs from PCDs and NoPCDs in the present study. Additionally, C3 + C16, which is the current auxiliary indicator for PCD NBS, was also incorporated. According to the ROC analysis (Table 2), C4 + C5 had the highest AUC (99.983%), followed by C4 + C5 + C14 (99.948%), C4 + C14 (99.887%), and C5 + C14 (98.958%). Regarding sensitivity, C4 + C5, C4 + C5 + C14, and C4 + C14 exhibited sensitivity of 100.000%, followed by C5 + C14 with sensitivity of 97.222%. For specificity, C4 + C5 had the highest value of 98.750%, followed by C4 + C5 + C14, C4 + C14 and C5 + C14 with specificities of 97.500%, 97.500% and 92.500%, respectively. Positive predictive values of C4 + C5, C4 + C5 + C14, C4 + C14 and C5 + C14 were 98.673%, 97.297%, 97.297% and 92.105%, respectively. The negative predictive values were 100.000% for C4 + C5, C4 + C5 + C14, and C4 + C14. C4 + C5 had the highest accuracy of 99.342%.

**Figure 3:**
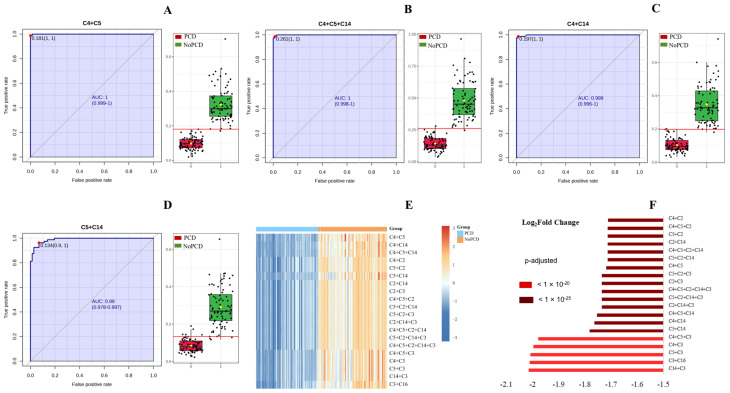
ROC curves and box plots of (**A**) C4 + C5, (**B**) C4 + C5 + C14, (**C**) C4 + C14, and (**D**) C5 + C14. The notch indicates the 95% confidence interval around the median of each group. The optimal cutoff is indicated with a horizontal red line on the boxplot. The mean concentration of each group is indicated with a yellow diamond. (**E**) cluster heatmap and (**F**) fold changes of specific various acylcarnitines summations in dried blood specimens from PCDs and NoPCDs. PCD, primary carnitine deficiency; ROC, receiver operator characteristic; AUC, area under the curve; C2, acetylcarnitine; C3, propionylcarnitine; C4, butyrylcarnitine; C5, isovalerylcarnitine; C14, myristoylcarnitine; C16, palmitoylcarnitine.

The ROC curves and the corresponding box plots of C4 + C5, C4 + C5 + C14, C4 + C14 and C5 + C14 are shown in Figure 3. ROC curves of the other various acylcarnitines summations are shown in Supplementary Figure S3. The notches of box plots for C4 + C5 had the least overlap (Figure 3A), indicating the best classification power for PCDs and NoPCDs. Figure 3E,F show a heatmap and the fold changes of the various acylcarnitines summations in PCDs and NoPCDs. C4 + C5, C4 + C5 + C14 and C4 + C14 exhibited the most significant changes (*q* value < 10^−25^). C3 + C16 showed a *q* value < 10^−20^.

**Figure 4:**
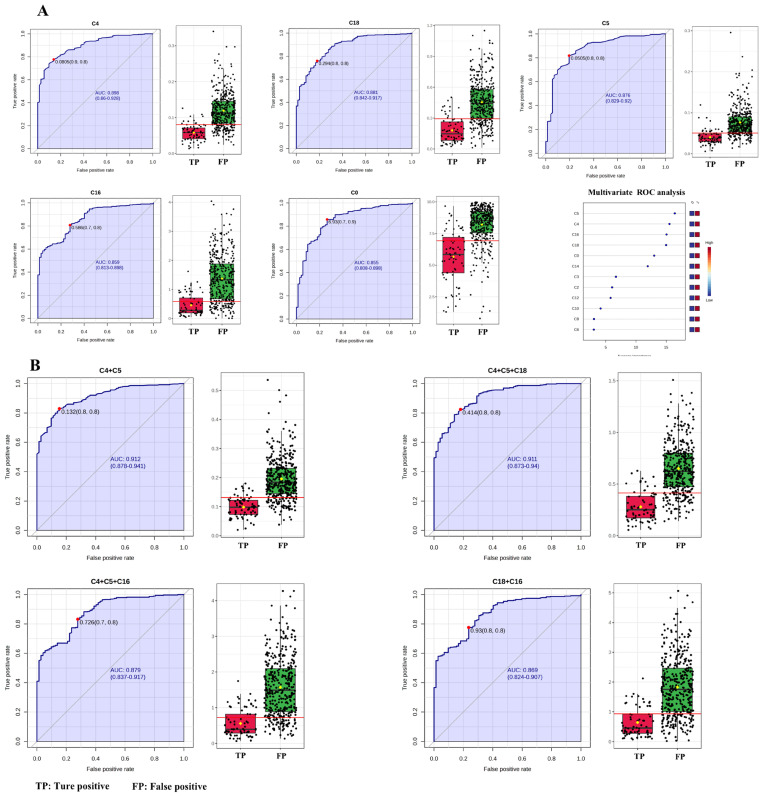
ROC curves and corresponding box plots between false-positive subjects and true-positive patients. (**A**) Univariate ROC curve analysis and multivariate ROC curve-based exploratory analysis. (**B**) C4 + C5, C4 + C5 + C18, C4 + C14 + C16 and C18 + C16. Each dots represents one individual sample. C4, butyrylcarnitine; C5, isovalerylcarnitine; C16, palmitoylcarnitine; C18, octadecylcarnitine.

To further verify the utility of C4 + C5 in NBS for PCD, ROC analysis between false-positive subjects from NBS data and the true-positive patients was performed. Univariate ROC curve analysis revealed that AUC was highest for C4 (89.608%), followed by C18 (87.993%), C5 (87.420%), C16 (85.873%) and C0 (85.457%) (Table 3 and Figure 4A). Multivariate exploratory ROC analysis also showed that C5, C4, C16 and C18 were the top-ranked features (Figure 4A). Therefore, the performances of C4 + C5, C4 + C5 + C18, C4 + C5 + C16 and C18 + C16 between false-positive and true-positive subjects were evaluated (Table 4 and Figure 4B). The ROC curves and the corresponding box plots are shown in Figure 4B. According to the ROC analysis (Table 4), C4 + C5 and C4 + C5 + C18 had AUCs of 91.052% and 91.054%, respectively, followed by C4 + C5 + C16 (87.777%) and C18 + C16 (86.770%). Regarding sensitivity, C4 + C5 + C16 exhibited the highest value (88.295%), followed by C4 + C5 (82.952%), C4 + C5 + C18 (78.880%), and C18 + C16 (58.015%). For specificity, C18 + C16 had the highest value of 97.222%, followed by C4 + C5 + C18, C4 + C5 and C4 + C5 + C16 with specificities of 86.111%, 84.722% and 68.056%, respectively. Positive predictive values of C4 + C5, C4 + C5 + C18, C4 + C5 + C16 and C18 + C16 were 96.736%, 96.875%, 93.784% and 99.130%, respectively. The negative predictive values were fairly low (<60.000%) for the four indicators. C4 + C5 and C4 + C5 + C16 had accuracies of 83.226% and 85.161%, respectively, followed by C4 + C5 + C18 (80.000%) and C18 + C16 (64.086%). C4 + C5 exhibited pretty good performances in AUC, sensitivity, specificity, positive predictive value and accuracy.

**Figure 5:**
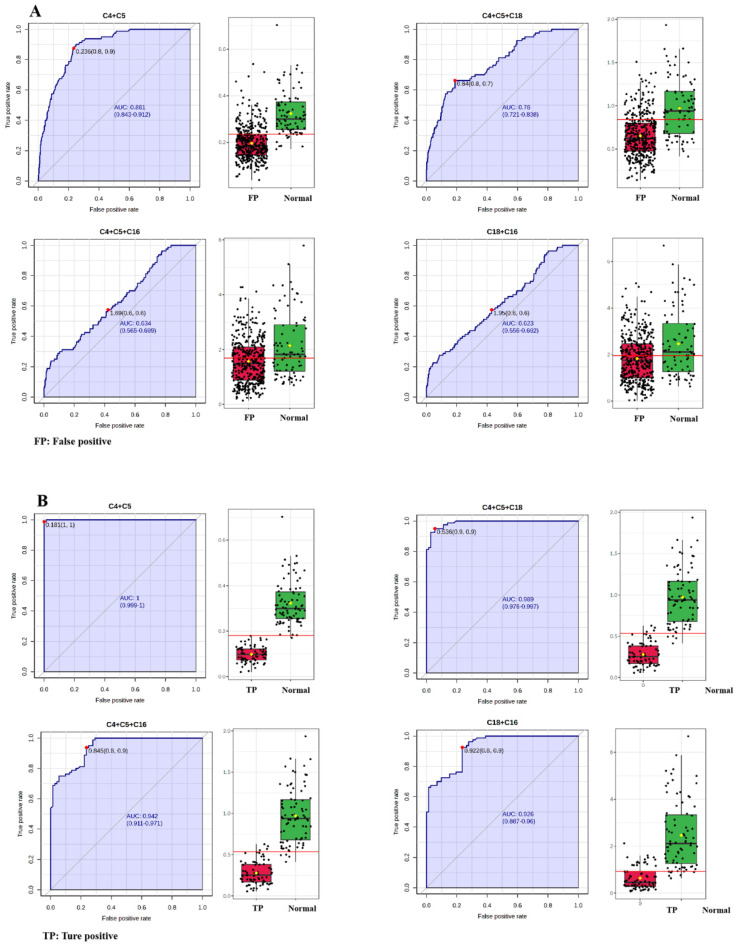
ROC analysis between false-positive subjects and those with normal C0 values (**A**). ROC analysis between true-positive patients and those with normal C0 values (**B**). Each dots represents one individual sample. C4, butyrylcarnitine; C5, isovalerylcarnitine; C16, palmitoylcarnitine; C18, octadecylcarnitine.

ROC analysis between false-positive subjects and those with normal C0 values was also performed for C4 + C5, C4 + C5 + C18, C4 + C5 + C16 and C18 + C16 (Table 4 and Figure 5A). C4 + C5 had the highest AUC of 88.034%, followed by C4 + C5 + C18 (77.861%), C4 + C5 + C16 (63.386%), and C18 + C16 (62.056%), respectively. This result indicated that C4 + C5 was the best indicator in differentiating false-positive subjects from normal ones. Additionally, ROC analysis between true-positive patients and those with normal C0 values for C4 + C5, C4 + C5 + C18, C4 + C5 + C16 and C18 + C16 also showed that the C4 + C5 exhibited the best performances in discriminating true-positive patients from normal ones (Table 4 and Figure 5B). AUC with the optimal cutoff was highest for C4 + C5 (99.983%), followed by C4 + C5 + C18 (98.880%), C4 + C5 + C16 (94.045%), and C18 + C16 (92.561%). The sensitivity, specificity, NPV, PPV, and accuracy indexes for C4 + C5 were 100.000%, 98.750%, 100.000%, 98.630%, and 99.342%, respectively (Table 4). This analysis evidenced that the screened C4 + C5 was a credible auxiliary diagnostic indicator for PCDs.

Table 5 shows the diagnostic performances of the C4 + C5, C4 + C5 + C14, C4 + C14 and C5 + C14 summations using optimal cutoffs determined by ROC analysis for PCD. The C4 + C5 summation yielded a false-positive result in only one patient. The C4 + C5 + C14 and C4 + C14 summations yielded false-positive results in two patients. There were no false-negative results in the C4 + C5, C4 + C5 + C14, C4 + C14 summation.

### 3.4. Validation and Application of Free Carnitine and C4 + C5 Summation in PCD NBS

Based on the above results, in this part, an additional NBS cohort program in Xinhua Hospital from 2021 to 2024 was utilized to assess the performance of the C0 and C4 + C5 summation in discriminating PCDs from NoPCDs. As shown in Table 6, the additional C4 + C5 summation (<0.181 μmol/L) utilization could elevate PPV from 0.75% to 1.54%, significantly reducing the false positives recalled (256 to 123). Among the 400 screening positives for PCD, up to 205 presented C4 + C5 summation above 0.181 µM. No one was a PCD patient in this population.

## 4. Discussion

PCD screening in neonates has been widely implemented across the world [2,29,30,31]. However, NBS for PCD is well known to have an unsatisfactory PPV, ranging from 1.6% to 4.7% [31,32]. If the C0 concentration is low at the initial screening, a second DBS sample is collected, and newborns are only referred when the low C0 concentration persists [17]. However, this workflow comes at the cost of timeliness. Initial DBSs are usually obtained 72–170 h after birth, and, in the event that a second test is requested, there is a further delay of 2–3 days before a newborn is referred. Additionally, false-positive outcomes in NBS could cause unnecessary anxieties for parents [33,34]. Hence, markers that have the best performances in sensitivity and specificity should be clarified to significantly reduce the false positives in PCD screening. In the present study, the levels of specific acylcarnitines and C0 were first compared in the 152 DBSs. In addition to C0, C4, C2 and C5 were also the most significantly downregulated markers (*q* < 10^−25^). Furthermore, the ROC analysis also showed that decreased C4, C2 and C5 levels exhibited the highest discrimination value (AUC > 0.980). Multivariate exploratory ROC analysis based on three algorithms (SVM, RF and PLS-DA) was performed to further screen the most reasonable acylcarnitines signatures, which could be utilized as auxiliary quantitative diagnostic indicators for PCD. The ROC analysis showed that the top three acylcarnitines (C4, C2 and C5, Figure 2) presented the highest discrimination value (AUC = 0.999–1). Next, C4 + C5, C4 + C2, C2 + C5 and C4 + C2 + C5 summations were evaluated in the 72 PCDs. Decreased C4 + C5 levels exhibited the highest discrimination value (AUC > 0. 999).

Considering the clinical symptoms of PCD and the accessibility of effective medical management, both false-positive and false-negative results should be prevented. Table 5 summarizes the performance of the C4 + C5 with the optimal cutoff applied to 72 PCDs and 80 NoPCDs. C4 + C5 yielded only one false-positive and no false-negative results. Furthermore, the performance of the C4 + C5 biomarker was evaluated in an additional NBS cohort including 85,404 infants. When using C0 < 10 µmol/L with C4 + C5 < 0.181 µmol/L, the PPV increased to 1.54% (3/195). It is worth noting that 92 cases with mildly decreased C0 (9.0~10.0 µmol/L) and C4 + C5 > 0.181 µmol/L were identified, and no abnormalities were found in 67 of them upon repeat testing (Table 6). Those with mildly decreased C0 but C4 + C5 > 0.181 µmol/L are less likely to be PCD patients. Therefore, in the screening system, adding the screening criteria C4 + C5 < 0.181 µmol/L can improve the positive predictive value, reduce the false-positive rate, and potentially help differentiate secondary carnitine deficiency, thereby improving the performance of newborn screening.

We should also pay attention to false negatives in PCD neonatal screening [35]. In the NBS cohort study, two PCD cases had a C0 value of 8.525 µmol/L and 8.937 µmol/L at the initial screening, which is very easy to miss. However, the corresponding C4 + C5 values were 0.121 µmol/L and 0.104 µmol/L, respectively. Our previous research on “Genomic Sequencing as a First-Tier Screening Test and Outcomes of Newborn Screening” also confirmed one PCD patient whose C0 and C4 + C5 values were 8.330 µmol/L and 0.120 µmol/L, respectively [36]. Therefore, it is important to pay attention to the recall of full-term neonates with a C0 critical value and C4 + C5 < 0.181 µmol/L to reduce the rate of missed diagnosis. Even if the neonatal screening is negative, if the neonate is clinically suspected of PCD, it still needs to be differentiated using tandem mass spectrometry. To further validate the performance of C4 + C5 < 0.181 µmol/L as an additional screening indicator, retrospective analysis was conducted on newborns recruited for PCD screening from 2003 to 2024 in Xinhua Hospital. One false-negative case (male) was reported, delivered at 38 weeks of gestation, weighing 3650 g at birth in 2016. Biochemical NBS screening showed that the C0 level was 11.637 µmol/L, not diagnosed with PCD in 2016. However, in 2019, this case exhibited clinical symptoms and *SLC22A5* gene mutation analysis showed two variants (c.760C > T; c.1400C > G). And the initial screening mass data in 2016 showed that C4 + C5 was 0.111 µmol/L. This false-negative case would not be missed if the sum of C4 + C5 was added to the NBS program. Based on the above results, the sum of C4 + C5 could be used as an effective auxiliary diagnostic indicator for PCDs with the lowest increase in false-positive and false-negative rates.

The added benefit of acylcarnitine summation in NBS has been reported previously in Guangzhou Newborn Screening Center, China. This research demonstrated that adding the quantitative biomarker C3 + C16 < 2 µmol/L into the newborn screening program could improve the PCD screen performance. However, in the present study population, using C3 + C16 < 2 µmol/L yielded 17 false positives and 5 false negatives (Table 5). Although we utilized the optimal cutoff of C3 + C16 determined by ROC analysis (1.839 µmol/L), there were up to 12 false positives and 6 false negatives, respectively. The performance is obviously lower than C4 + C5.

We acknowledge the limitations of using the C4 + C5 summation as an additional discriminative tool for PCD. In the Genomic Sequencing Program, with 29,989 infants recruited from eight NBS centers throughout China, one PCD patient was not detected by genetic NBS but revealed via tandem mass spectrometry. This patient exhibited a C0 value of 6.65 µmol/L. The sum of C4 + C5 was 0.190 µmol/L, slightly higher than the optimal cutoff (0.181 µmol/L) determined in the present study. Larger studies are necessary to validate the C4 + C5 threshold. The other limitation of the present study was the lack of reference values for the NBS program in PCD, except the currently used C0 level. The sum of (C4 + C5)’s performance must be further validated using nationwide NBS data.

## 5. Conclusions

In this study, we performed a comprehensive evaluation of the diagnostic performances of specific acylcarnitine levels and various acylcarnitine summations using two PCD cohorts. The utilization of C0 < 10 µmol/L with C4 + C5 < 0.181 µmol/L can effectively discriminate between true- and false-positive referrals for PCD, reducing time to diagnosis and mitigating the negative effects of a false-positive referral. The performance of C0 plus C4 + C5 will be further evaluated in the retrospective and prospective nationwide studies for PCD. The optimal and appropriate reference ranges can be established, improving differential PCD diagnosis with significant reductions in the false positivity and false negativity rates.

## Figures and Tables

**Table 1:** ROC analysis of free carnitine and specific acylcarnitines levels in dried blood specimens.

Characteristic	Free Carnitine and Acylcarnitine											
	C0	C2	C3	C4	C5	C6	C8	C10	C12	C14	C16	C18
AUC (%)	100.000	98.715	96.753	99.792	98.620	86.623	92.274	95.200	88.168	95.738	91.319	95.095
Best Cutoff Value (μmol/L)	11.854	10.973	0.882	0.111	0.063	0.043	0.035	0.042	0.046	0.077	0.583	0.305
Sensitivity (%)	100.000	98.611	95.833	98.611	94.444	76.389	75.000	81.944	83.333	91.667	72.222	83.333
Specificity (%)	100.000	95.000	85.000	98.750	100.000	82.500	97.500	96.250	78.750	87.500	95.000	91.250
Negative Predictive Value (%)	100.000	98.701	95.775	98.750	95.238	79.518	81.250	85.556	84.000	92.105	79.167	85.882
Positive Predictive Value (%)	100.000	94.667	85.185	98.611	100.000	79.710	96.429	95.161	77.922	86.842	92.857	89.552
Accuracy (%)	100.000	96.711	90.132	98.684	97.368	79.605	86.842	89.474	80.921	89.474	84.211	87.500

AUC, area under the curve; C0, carnitine; C2, acetylcarnitine; C3, propionylcarnitine; C4, butyrylcarnitine; C5, isovalerylcarnitine; C6, caproylcarnitine; C8, caprylylcarnitine; C10, actinylcarnitine, C12, lauroylcarnitine; C14, myristoylcarnitine; C16, palmitoylcarnitine; C18, octadecylcarnitine.

**Table 2:** ROC analysis of specific various acylcarnitines summations in dried blood specimens.

Acylcarnitines Summations	AUC (%)	Best Cutoff Value (μmol/L)	Sensitivity (%)	Specificity (%)	Negative Predictive Value (%)	Positive Predictive Value (%)	Accuracy (%)
C4 + C5	99.983	0.181	100.000	98.750	100.000	98.630	99.342
C4 + C5 + C14	99.948	0.281	100.000	97.500	100.000	97.297	98.684
C4 + C14	99.887	0.201	100.000	97.500	100.000	97.297	98.684
C5 + C14	98.958	0.151	97.222	92.500	97.368	92.105	94.737
C4 + C5 + C2 + C14 + C3	98.872	11.792	98.611	96.250	98.718	95.946	97.368
C5 + C2 + C3	98.854	11.598	98.611	96.250	98.718	95.946	97.368
C4 + C5 + C2 + C14	98.837	11.201	98.611	95.000	98.701	94.667	96.711
C5 + C2 + C14 + C3	98.837	11.691	98.611	96.250	98.718	95.946	97.368
C2 + C3	98.819	11.502	98.611	96.250	98.718	95.946	97.368
C4 + C5 + C2	98.811	11.117	98.611	95.000	98.701	94.667	96.711
C2 + C14 + C3	98.811	11.595	98.611	96.250	98.718	95.946	97.368
C5 + C2	98.750	11.019	98.611	95.000	98.701	94.667	96.711
C5 + C2 + C14	98.750	11.099	98.611	95.000	98.701	94.667	96.711
C4 + C2	98.733	11.070	98.611	95.000	98.701	94.667	96.711
C2 + C14	98.733	11.053	98.611	95.000	98.701	94.667	96.711
C4 + C5 + C3	97.986	0.764	84.722	100.000	87.912	100.000	92.763
C4 + C3	97.457	0.984	95.833	87.500	95.890	87.342	91.447
C5 + C3	97.274	0.937	95.833	88.750	95.946	88.462	92.105
C14 + C3	97.083	0.961	95.833	86.250	95.833	86.250	90.789
C3 + C16	96.215	1.839	91.667	85.000	91.892	84.615	88.158

ROC, receiver operator characteristic; AUC, area under the curve; C2, acetylcarnitine; C3, propionylcarnitine; C4, butyrylcarnitine; C5, isovalerylcarnitine; C14, myristoylcarnitine; C16, palmitoylcarnitine.

**Table 3:** ROC analysis of free carnitine and specific acylcarnitines levels in dried blood specimens from true-positive and false-positive subjects.

Characteristic	Free Carnitine and Acylcarnitine											
	C4	C18	C5	C16	C0	C14	C3	C2	C10	C12	C8	C6
AUC (%)	89.608	87.993	87.420	85.873	85.457	85.314	80.928	74.046	73.420	66.622	63.965	60.888
Best Cutoff Value (μmol/L)	0.086	0.224	0.051	1.068	6.934	0.074	0.574	7.763	0.047	0.052	0.035	0.061
Sensitivity (%)	73.791	88.550	81.679	61.069	85.751	64.122	68.957	63.359	46.819	47.837	48.346	23.664
Specificity (%)	90.278	69.444	80.556	93.056	73.611	91.667	80.556	80.556	90.278	88.889	75.000	94.444
Negative Predictive Value (%)	38.690	52.632	44.615	30.455	48.624	31.884	32.222	28.713	23.723	23.792	21.012	18.478
Positive Predictive Value (%)	97.643	94.054	95.821	97.959	94.663	97.674	95.088	94.677	96.335	95.918	91.346	95.876
Accuracy (%)	76.344	85.591	81.505	66.022	83.871	68.387	70.753	66.022	53.548	54.194	52.473	34.624

AUC, area under the curve; C0, carnitine; C2, acetylcarnitine; C3, propionylcarnitine; C4, butyrylcarnitine; C5, isovalerylcarnitine; C6, caproylcarnitine; C8, caprylylcarnitine; C10, actinylcarnitine, C12, lauroylcarnitine; C14, myristoylcarnitine; C16, palmitoylcarnitine; C18, octadecylcarnitine.

**Table 4:** ROC analysis of C4 + C5, C4 + C5 + C18, C4 + C5 + C16 and C18 + C16 in dried blood specimens from true-positive (TP), false-positive (FP) and normal subjects.

Indicator: C4 + C5				Indicator: C4 + C5 + C18			
Characteristic	TP vs. FP	FP vs. Normal	TP vs. Normal	Characteristic	TP vs. FP	FP vs. Normal	TP vs. Normal
AUC (%)	91.052	88.034	99.983	AUC (%)	91.054	77.861	98.880
Best Cutoff Value (μmol/L)	0.132	0.233	0.181	Best Cutoff Value (μmol/L)	0.441	0.841	0.573
Sensitivity (%)	82.952	90.000	100.000	Sensitivity (%)	78.880	66.250	92.500
Specificity (%)	84.722	75.064	98.750	Specificity (%)	86.111	81.170	97.222
Negative Predictive Value (%)	47.656	97.360	100.000	Negative Predictive Value (%)	42.759	92.197	92.105
Positive Predictive Value (%)	96.736	42.353	98.630	Positive Predictive Value (%)	96.875	41.732	97.368
Accuracy (%)	83.226	77.590	99.342	Accuracy (%)	80.000	78.647	94.737
Indicator: C4 + C5 + C16				Indicator: C18 + C16			
Characteristic	TP vs. FP	FP vs. Normal	TP vs. Normal	Characteristic	TP vs. FP	FP vs. Normal	TP vs. Normal
AUC (%)	87.777	63.386	94.045	AUC (%)	86.770	62.056	92.561
Best Cutoff Value (μmol/L)	0.617	2.760	0.737	Best Cutoff Value (μmol/L)	1.539	3.301	0.922
Sensitivity (%)	88.295	28.750	98.750	Sensitivity (%)	58.015	27.500	92.500
Specificity (%)	68.056	90.840	72.222	Specificity (%)	97.222	91.603	76.389
Negative Predictive Value (%)	51.579	86.232	98.113	Negative Predictive Value (%)	29.787	86.124	90.164
Positive Predictive Value (%)	93.784	38.983	79.798	Positive Predictive Value (%)	99.130	40.000	81.319
Accuracy (%)	85.161	80.338	86.184	Accuracy (%)	64.086	80.761	84.868

C4, butyrylcarnitine; C5, isovalerylcarnitine; C16, palmitoylcarnitine; C18, octadecylcarnitine.

**Table 5:** Assesment of the C4 + C5, C4 + C5 + C14, C4 + C14 and C5 + C14 summations using optimal cutoffs determined by ROC analysis.

Indicator: C4 + C5 < 0.181 μmol/L					Indicator: C4 + C5 + C14 < 0.281 μmol/L				
PCD	Data Available	Positive	Negative	Sensitivity	PCD	Data Available	Positive	Negative	Sensitivity
	72	72	0	1		72	72	0	1
NoPCD	Data Available	Positive	Negative	Specificity	NoPCD	Data Available	Positive	Negative	Specificity
	80	1	79	0.988		80	2	78	0.975
PCD + NoPCD	Positive predictive value		Negative predictive value		PCD + NoPCD	Positive predictive value		Negative predictive value	
	0.986		1			0.973		1	
Indicator: C4 + C14 < 0.201 μmol/L					Indicator: C5 + C14 < 0.151 μmol/L				
PCD	Data Available	Positive	Negative	Sensitivity	PCD	Data Available	Positive	Negative	Sensitivity
	72	72	0	1		72	70	2	0.972
NoPCD	Data Available	Positive	Negative	Specificity	NoPCD	Data Available	Positive	Negative	Specificity
	80	2	78	0.975		80	6	74	0.925
PCD + NoPCD	Positive predictive value		Negative predictive value		PCD + NoPCD	Positive predictive value		Negative predictive value	
	0.973		1			0.923		0.974	
Indicator: C3 + C16 < 1.839 μmol/L					Indicator: C3 + C16 < 2.000 μmol/L				
PCD	Data Available	Positive	Negative	Sensitivity	PCD	Data Available	Positive	Negative	Sensitivity
	72	66	6	0.917		72	67	5	0.931
NoPCD	Data Available	Positive	Negative	Specificity	NoPCD	Data Available	Positive	Negative	Specificity
	80	12	68	0.850		80	17	63	0.788
PCD + NoPCD	Positive predictive value		Negative predictive value		PCD + NoPCD	Positive predictive value		Negative predictive value	
	0.846		0.919			0.798		0.926	

C3, propionylcarnitine; C4, butyrylcarnitine; C5, isovalerylcarnitine; C14, myristoylcarnitine; C16, palmitoylcarnitine.

**Table 6:** Application of the C0 level and C4 + C5 summation using optimal cutoffs determined by ROC analysis in NBS from 2021 to 2024.

Indicators (μmol/L)	Screening Positive (N)	Recall (N)	Recall Negative (N)	Recall Positive (N)	Confirmed (N)	Positive Predictive Value (%)
C0 < 10	400	376	256	120	3	0.75
C0 < 10 & C4 + C5 < 0.181	195	185	123	62	3	1.54
C0 < 10 & C4 + C5 > 0.181	205	188	133	55	0	NA
9 < C0 < 10 & C4 + C5 > 0.181	92	88	67	21	0	NA
8.5 < C0 < 10 & C4 + C5 < 0.181	83	76	58	18	2	NA

ROC, receiver operator characteristic; C0, carnitine; C4, butyrylcarnitine; C5, isovalerylcarnitine.

## Data Availability

The datasets used and analyzed in this study are available from the corresponding author on reasonable request.

## Supplementary Materials

The following supporting information can be downloaded at: https://www.mdpi.com/article/10.3390/ijns11020047/s1, Figure S1: Performance of acylcarnitines in identifying patients with PCD; Figure S2: Box plots of acylcarnitines summations in dried blood specimens; Figure S3: ROCs of various acylcarnitines summations in dried blood specimens to diagnose PCDs; Table S1: Genetic variants of the PCD patients in the study cohort; Table S2: Levels of free carnitine and specific acylcarnitines in dried blood specimens (μmol/L); Table S3: Levels of various acylcarnitines summations in dried blood specimens (μmol/L).
