# Supplementary material for: Assessment and Application of Acylcarnitines Summations as Auxiliary Quantization Indicator for Primary Carnitine Deficiency
Source: Int J Neonatal Screen. 2025 Jun 19;11(2):47. doi: 10.3390/ijns11020047 (PMC12192769; doi:10.3390/ijns11020047)
Supplement: Supplementary file 1 [file IJNS-11-00047-s001.zip › IJNS-3611136-supplementary.pdf]

Assessment and application of acylcarnitines summations as auxiliary quantization indicator for primary carnitine deficiency

Haijuan Zhi [a,†], Siyu Chang [a,†], Ting Chen [a], Lili Liang [a], Wenjuan Qiu [a], Huiwen Zhang [a], Xuefan Gu [a] and Lianshu Han [a,*]

[a] Department of Pediatric Endocrinology and Genetic Metabolism, Xinhua Hospital, Shanghai Institute for Pediatric Research, Shanghai Jiaotong University School of Medicine, Shanghai 200092, China

Correspondence: hanlianshu@xinhuamed.com.cn (L.H.)

[†] These authors contributed equally to this work.

Supplementary Figure S1

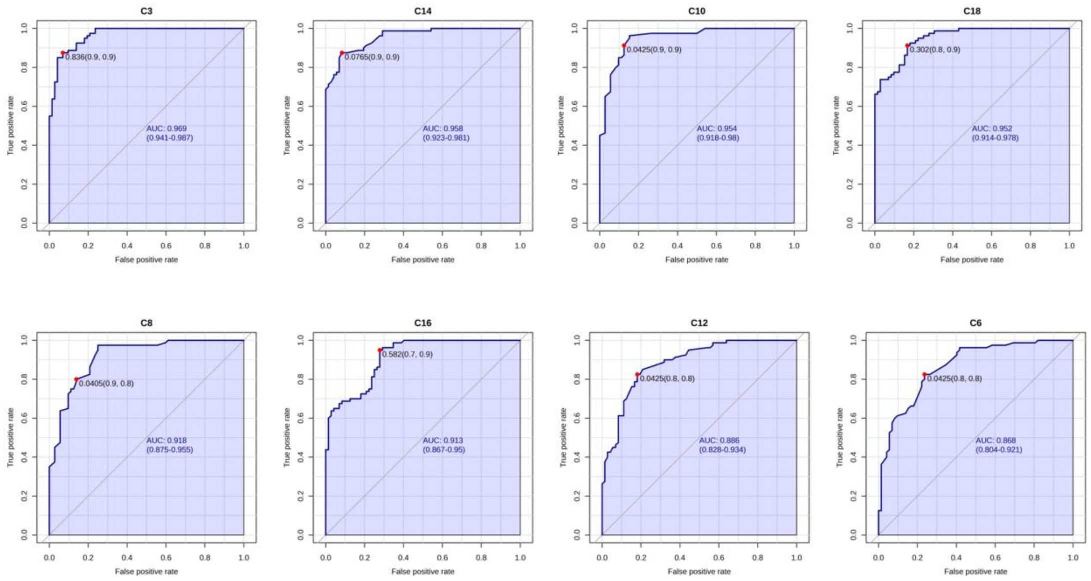

Supplementary Figure S1. Performance of acylcarnitines in identifying patients with PCD. ROC, receiver operator characteristic; AUC, area under the curve; C3, propionylcarnitine; C6, caproylcarnitine; C8, caprylylcarnitine; C10, actinylcarnitine, C12, lauroylcarnitine; C14, myristoylcarnitine; C16, palmitoylcarnitine; C18, octadecylcarnitine

Supplementary Figure S2

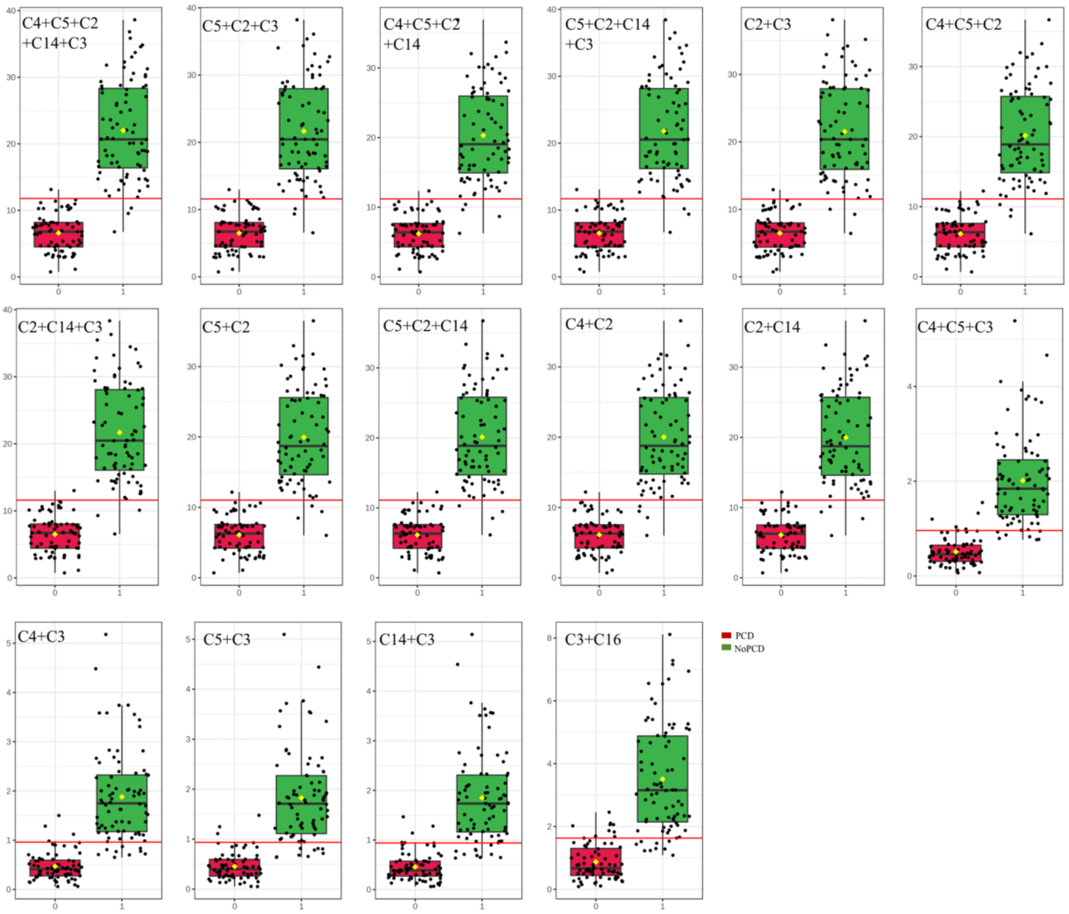

Supplementary Figure S2. Box plots of acylcarnitines summations in dried blood specimens. The optimal cutoff is indicated with a horizontal red line on the boxplot. The mean concentration of each group is indicated with a yellow diamond. C2, acetylcarnitine; C3, propionylcarnitine; C4, butyrylcarnitine; C5, isovalerylcarnitine; C14, myristoylcarnitine; C16, palmitoylcarnitine.

Supplementary Figure S3

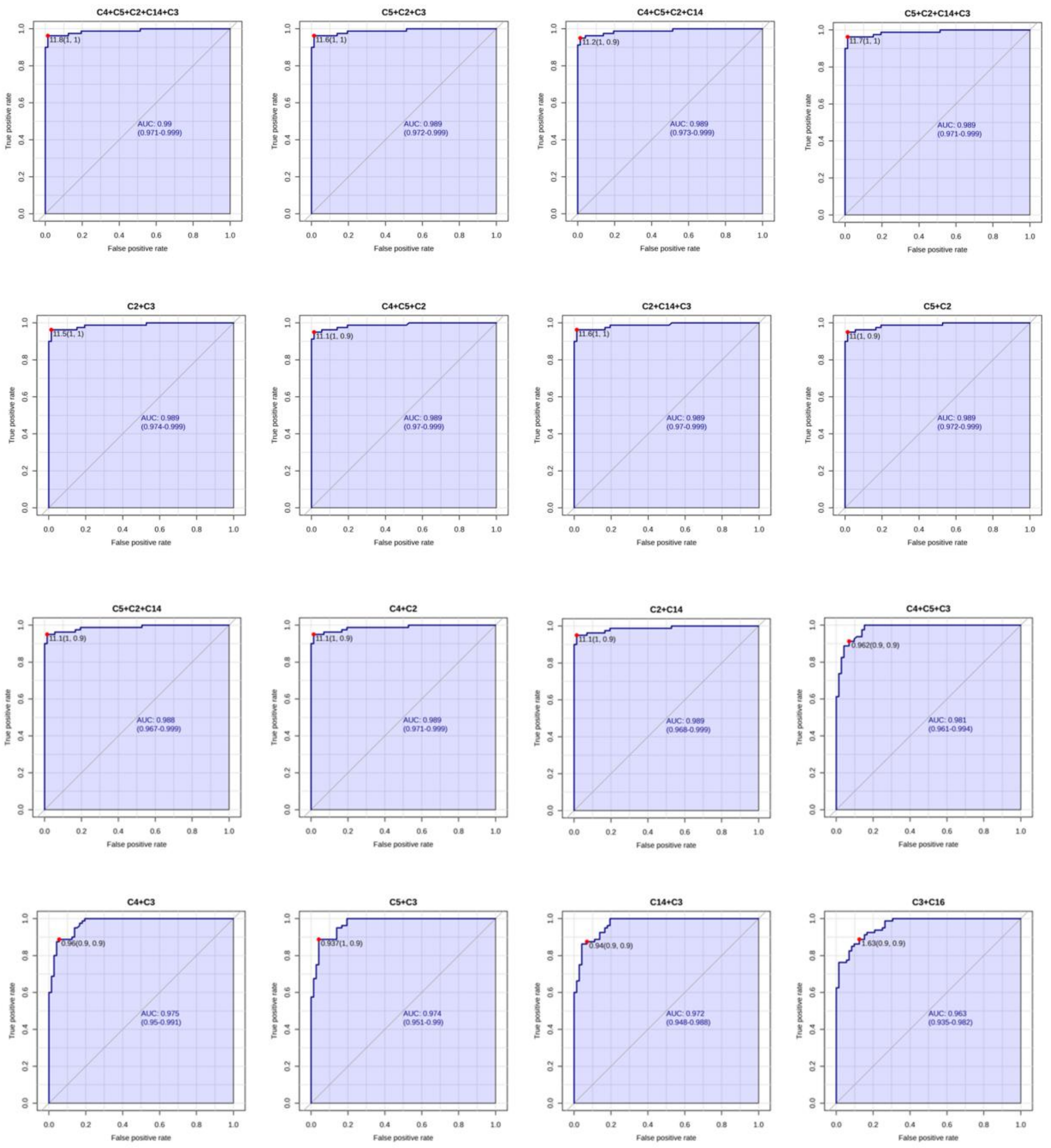

Supplementary Figure S3**.** ROCs of various acylcarnitines summations in dried blood specimens to diagnose PCDs. ROC, receiver operator characteristic; AUC, area under the curve; C2, acetylcarnitine; C3, propionylcarnitine; C4, butyrylcarnitine; C5, isovalerylcarnitine; C14, myristoylcarnitine; C16, palmitoylcarnitine.

Supplementary Table S1. Genetic variants of the PCD patients in the study cohort.

Case number	C0 (μmol/L)	Coding DNA change allele 1	Protein change allele 1	Coding DNA change allele 2	Protein change allele 2
1	4.391	c.1400C>G	p.S467C	c.1400C>G	p.S467C
2	4.243	c.137delC	p.P46Rfs*3	c.844C>T	p.R282X
3	6.266	c.497+1G>T		c.680G>A	p.R227H
4	6.221	c.1400C>G	p.S467G	c.1400C>G	p.S467G
5	2.313	c.951+2T>C		c.1400C>G	p.S467G
6	6.908	c.248G>A	p.R83H	c.248G>A	p.R83H
7	7.905	c.1199G>A	p.R400H	c.1400C>G	p.S467C
8	1.686	c.745_748del	p.F249Lfs*14	c.1319C>T	p.T440M
9	1.305	c.51C>G	p.F17L	c.1433C>T	p.P478L
10	5.946	c.427C>T	p.P143S	c.428C>T	p.P143L
11	4.492	c.41G>A	p.W14X	c.1400C>G	p.S467G
12	2.632	c.41G>A	p.W14X	c.1400C>G	p.S467C
13	5.459	c.14000C>G	p.S467C	c.14000C>G	p.S467C
14	4.392	c.760C>T	p.R254X	c.1195C>T	P.R399W
15	7.863	c.494A>G	p.D165G	c.1400C>G	p.S467C
16	2.570	c.51C>G	p.F17L	c.802delG	p.V268Cfs*28
17	5.960	c.51C>G	p.F17L	c.1400C>G	p.S467C
18	3.188	c.338G>A	p.C113Y	c.1400C>G	p.S467C
19	7.175	c.1400C>G	p.S467C	c.1526T>C	p.L509P
20	1.390	c.384dupT	p.V129Cfs*9	c.1267+2T>C	
21	1.757	c.497G>T	p.R166M	c.1400C>G	p.S467C
22	7.761	c.51C>G	p.F17L	c.51C>G	p.F17L
23	5.510	c.14000C>G	p.S467C	Not detected	
24	7.280	c.760C>T	p.R254X	Not detected	
25	5.270	c.1195C>T	p.R399W	c.850C>G	p.L284V
26	6.250	c.428C>T	p.P143L	c.839C>T	p.S280F
27	5.330	c.1400C>G	p.S467C	c.14000C>G	p.S467C
28	4.232	c.51C>G	p.F17L	c.1195C>T	p.R399W
29	6.394	c.51C>G	p.F17L	c.797C>T	p.P266L
30	8.002	c.338G>A	p.C113Y	c.797C>T	p.P266L
31	4.940	c.338G>A	p.C113Y	c.797C>T	p.P266L
32	5.309	c.51C>G	p.F17L	c. 1337T>G	p.L378R
33	5.160	c.51C>G	p.F17L	c. 1337T>G	p.L378R
34	9.082	c.428C>T	p.P143L	c.1400C>G	p.S467C
35	7.420	c.428C>T	p.P143L	c.1400C>G	p.S467C
36	3.166	c.51C>G	p.F17L	c.1400C>G	p.S467C
37	6.300	c.51C>G	p.F17L	c.1400C>G	p.S467C
38	6.862	c.338G>A	p.C113Y	c.1400C>G	p.S467C
39	4.720	c.338G>A	p.C113Y	c.1400C>G	p.S467C
40	8.096	c.51C>G	p.F17L	c.1445A>G	p.Y482C

41	6.320	c.51C>G	p.F17L	c.1445A>G	p.Y482C
42	2.729	c.51C>G	p.F17L	c.760C>T	p.R254X
43	5.470	c.51C>G	p.F17L	c.760C>T	p.R254X
44	6.908	c.248G>A	p.R83H	c.248G>A	p.R83H
45	3.870	c.248G>A	p.R83H	c.248G>A	p.R83H
46	7.905	c.1199G>A	p.R400H	c.1400C>G	p.S467C
47	8.515	c.1199G>A	p.R400H	c.1400C>G	p.S467C
48	5.642	c.51C>G	p.F17L	c.844C>T	p.R282X
49	5.960	c.51C>G	p.F17L	c.1400C>G	p.S467C
50	8.292	c.51C>G	p.F17L	c.1400C>G	p.S467C
51	4.399	c.494A>G	p.D165G	c.1400C>G	p.S467C
52	7.709	c.494A>G	p.D165G	c.1400C>G	p.S467C
53	8.893	c.136C>G	p.P46A	c.1400C>G	p.S467C
54	9.661	c.136C>G	p.P46A	c.1400C>G	p.S467C
55	6.414	c.1400C>G	p.S467C	c.1400C>G	p.S467C
56	6.763	c.338G>A	p.C113Y	c.1400C>G	p.S467C
57	4.640	c.407G>A	p.C136Y	c.1400C>G	p.S467C
58	5.593	c.51C>G	p.F17L	c.1400C>G	p.S467C
59	7.349	c.497+1G>T		c.680G>A	p.R227H
60	6.258	c.51C>G	p.F17L	c.797C>T	p.P266L
61	6.853	c.51C>G	p.F17L	c.1400C>G	p.S467C
62	5.786	c.51C>G	p.F17L	c.1400C>G	p.S467C
63	2.518	c.51C>G	p.F17L	c.51C>G	p.F17L
64	7.855	c.428C>T	p.P143L	c.1400C>G	p.S467C
65	4.007	c.51C>G	p.F17L	c.361C>T	p.Q121X
66	2.432	c.51C>G	p.F17L	c.385 insT	
67	5.786	c.760C>T	p.R254X	c.1400C>G	p.S467C
68	5.195	c.396G>A	p.W132X	c.1400C>G	p.S467C
69	3.243	c.51C>G	p.F17L	c.248G>T	p.R83L
70	5.891	Not Detected		Not Detected	
71	8.937	c.51C>G	p.F17L	c.1237G>A	p.V413I
72	8.525	c.1400C>G	p.S467C	c.1400C>G	p.S467C

C0, carnitine; PCD, primary carnitine deficiency

Supplementary Table S2. Levels of free carnitine and specific acylcarnitines in dried blood specimens (μmol/L).

Case ID	C0	C2	C3	C4	C5	C6	C8	C10	C12	C14	C16	C18
P1	4.391	2.969	0.274	0.040	0.014	0.009	0.019	0.024	0.013	0.017	0.188	0.071
P2	4.243	5.074	0.186	0.059	0.042	0.070	0.032	0.038	0.021	0.016	0.084	0.072
P3	6.266	4.828	0.207	0.031	0.028	0.033	0.019	0.019	0.024	0.020	0.170	0.073
P4	6.221	4.958	0.168	0.021	0.032	0.038	0.014	0.022	0.018	0.029	0.210	0.096
P5	2.313	2.855	0.126	0.016	0.022	0.018	0.022	0.029	0.024	0.023	0.104	0.049
P6	6.908	7.385	0.560	0.051	0.034	0.024	0.032	0.026	0.032	0.031	0.225	0.102
P7	7.905	7.488	0.512	0.067	0.088	0.055	0.037	0.042	0.032	0.045	0.253	0.114
P8	1.686	3.615	0.186	0.029	0.030	0.047	0.022	0.026	0.015	0.023	0.078	0.032
P9	1.305	4.407	0.148	0.051	0.030	0.039	0.030	0.027	0.029	0.021	0.095	0.052
P10	5.946	4.409	0.285	0.092	0.039	0.023	0.026	0.024	0.024	0.018	0.092	0.039
P11	4.492	7.059	0.142	0.044	0.119	0.036	0.036	0.024	0.021	0.054	0.207	0.121
P12	2.632	4.481	0.109	0.041	0.026	0.024	0.028	0.015	0.019	0.022	0.113	0.054
P13	5.459	5.860	0.442	0.057	0.031	0.016	0.034	0.022	0.016	0.027	0.197	0.084
P14	4.392	6.488	0.207	0.046	0.028	0.026	0.025	0.022	0.021	0.040	0.328	0.170
P15	7.863	4.225	0.410	0.074	0.029	0.014	0.040	0.045	0.015	0.018	0.282	0.186
P16	2.570	2.824	0.112	0.038	0.020	0.014	0.012	0.029	0.019	0.017	0.078	0.024
P17	5.960	7.371	0.260	0.041	0.055	0.027	0.034	0.028	0.036	0.033	0.238	0.104
P18	3.188	4.336	0.104	0.046	0.027	0.016	0.026	0.013	0.026	0.015	0.233	0.066
P19	7.175	7.193	0.313	0.054	0.046	0.035	0.035	0.023	0.021	0.021	0.243	0.200
P20	1.390	1.069	0.046	0.017	0.008	0.030	0.005	0.004	0.008	0.021	0.042	0.031
P21	1.757	0.686	0.045	0.014	0.006	0.009	0.005	0.005	0.007	0.017	0.061	0.047
P22	7.761	4.272	0.331	0.039	0.027	0.016	0.016	0.014	0.013	0.029	0.153	0.091
P23	5.510	2.690	0.210	0.050	0.030	0.010	0.010	0.010	0.010	0.020	0.170	0.070

P24	7.280	9.720	0.830	0.070	0.050	0.030	0.060	0.030	0.020	0.070	0.210	0.290
P25	5.270	3.110	0.250	0.060	0.050	0.010	0.010	0.020	0.020	0.030	0.360	0.170
P26	6.250	3.120	0.290	0.070	0.030	0.020	0.060	0.070	0.020	0.030	0.290	0.180
P27	5.330	2.920	0.260	0.050	0.030	0.010	0.020	0.020	0.020	0.030	0.320	0.120
P28	4.232	4.280	0.131	0.044	0.028	0.034	0.018	0.026	0.021	0.031	0.236	0.081
P29	6.394	3.395	0.374	0.049	0.023	0.015	0.021	0.026	0.011	0.024	0.252	0.132
P30	8.002	6.342	1.426	0.075	0.050	0.053	0.032	0.041	0.043	0.040	0.204	0.102
P31	4.940	5.780	0.310	0.060	0.020	0.030	0.030	0.020	0.030	0.040	0.510	0.190
P32	5.309	4.808	0.379	0.064	0.048	0.039	0.021	0.050	0.028	0.021	0.180	0.096
P33	5.160	9.300	0.640	0.060	0.040	0.050	0.020	0.040	0.040	0.080	0.820	0.340
P34	9.082	4.234	0.388	0.067	0.057	0.035	0.017	0.013	0.023	0.024	0.183	0.076
P35	7.420	8.980	1.030	0.090	0.080	0.060	0.030	0.050	0.090	0.110	1.030	0.400
P36	3.166	2.751	0.284	0.037	0.036	0.028	0.033	0.020	0.012	0.030	0.239	0.123
P37	6.300	9.500	0.880	0.070	0.040	0.040	0.040	0.040	0.030	0.070	1.140	0.370
P38	6.862	5.080	0.328	0.035	0.038	0.031	0.065	0.044	0.040	0.057	0.457	0.230
P39	4.720	10.190	0.780	0.080	0.060	0.040	0.050	0.060	0.040	0.050	0.680	0.240
P40	8.096	4.007	0.344	0.062	0.062	0.114	0.038	0.038	0.041	0.041	0.222	0.097
P41	6.320	6.060	0.440	0.050	0.030	0.040	0.030	0.030	0.040	0.050	0.530	0.170
P42	2.729	3.882	0.071	0.023	0.031	0.056	0.019	0.032	0.030	0.020	0.078	0.041
P43	5.470	10.641	0.570	0.075	0.028	0.067	0.034	0.040	0.059	0.062	1.107	0.418
P44	6.908	7.385	0.560	0.051	0.034	0.024	0.032	0.026	0.032	0.031	0.225	0.102
P45	3.870	2.500	0.480	0.040	0.020	0.020	0.010	0.020	0.030	0.040	0.520	0.180
P46	7.905	7.488	0.512	0.067	0.088	0.055	0.037	0.042	0.032	0.045	0.253	0.114
P47	8.515	12.115	0.802	0.084	0.044	0.051	0.043	0.049	0.068	0.101	1.258	0.271
P48	5.642	9.558	0.641	0.080	0.040	0.054	0.033	0.022	0.050	0.067	0.547	0.197
P49	5.960	7.371	0.260	0.041	0.055	0.027	0.034	0.028	0.036	0.033	0.238	0.104

P50	8.292	10.581	0.781	0.090	0.047	0.026	0.024	0.025	0.041	0.096	0.911	0.283
P51	4.399	3.101	0.218	0.059	0.031	0.017	0.010	0.012	0.010	0.022	0.115	0.048
P52	7.709	9.540	0.833	0.085	0.041	0.027	0.026	0.030	0.045	0.103	1.620	0.502
P53	8.893	2.805	0.187	0.059	0.036	0.022	0.022	0.031	0.024	0.039	0.129	0.064
P54	9.661	5.909	0.278	0.104	0.057	0.037	0.042	0.021	0.049	0.062	0.520	0.217
P55	6.414	9.599	0.504	0.071	0.045	0.042	0.046	0.032	0.077	0.058	0.494	0.322
P56	6.763	7.652	0.873	0.109	0.055	0.019	0.024	0.029	0.042	0.071	0.994	0.341
P57	4.640	6.340	0.400	0.070	0.050	0.020	0.010	0.030	0.030	0.040	0.890	0.410
P58	5.593	6.656	0.339	0.053	0.039	0.040	0.034	0.026	0.035	0.030	0.467	0.160
P59	7.349	7.243	0.427	0.078	0.055	0.055	0.032	0.024	0.072	0.064	0.962	0.471
P60	6.258	7.743	0.369	0.058	0.060	0.026	0.025	0.046	0.051	0.053	0.698	0.223
P61	6.853	7.306	0.526	0.104	0.038	0.041	0.032	0.035	0.031	0.047	0.582	0.258
P62	5.786	7.540	0.383	0.045	0.040	0.026	0.050	0.056	0.074	0.053	0.728	0.179
P63	2.518	7.120	0.247	0.084	0.047	0.067	0.066	0.045	0.035	0.055	0.379	0.121
P64	7.855	10.104	0.612	0.126	0.054	0.046	0.040	0.065	0.044	0.071	0.931	0.375
P65	4.007	7.158	0.236	0.033	0.025	0.016	0.024	0.031	0.024	0.047	0.279	0.134
P66	2.432	6.400	0.402	0.044	0.036	0.034	0.046	0.040	0.050	0.061	0.936	0.304
P67	5.786	9.401	0.436	0.066	0.048	0.057	0.033	0.036	0.071	0.067	0.921	0.364
P68	5.195	7.254	0.421	0.030	0.034	0.017	0.024	0.026	0.027	0.072	1.065	0.261
P69	3.243	2.579	0.268	0.026	0.017	0.011	0.011	0.014	0.016	0.056	0.375	0.174
P70	5.891	9.418	0.634	0.072	0.037	0.052	0.033	0.033	0.059	0.092	1.191	0.411
P71	8.937	6.128	0.525	0.064	0.040	0.028	0.029	0.034	0.028	0.051	0.925	0.273
P72	8.525	7.093	1.206	0.079	0.042	0.028	0.024	0.03	0.038	0.073	0.898	0.292
M_PCD	5.839	6.234	0.357	0.059	0.038	0.030	0.030	0.029	0.030	0.040	0.280	0.147
Q1_PCD	4.392	4.171	0.231	0.041	0.030	0.020	0.020	0.022	0.020	0.024	0.195	0.083
Q3_PCD	7.201	7.488	0.525	0.071	0.048	0.041	0.034	0.038	0.040	0.059	0.706	0.264

NP1	33.562	19.073	1.422	0.159	0.151	0.048	0.054	0.059	0.050	0.090	0.917	0.476
NP2	28.701	16.709	1.148	0.189	0.125	0.047	0.088	0.125	0.048	0.061	1.090	0.614
NP3	40.289	23.317	0.998	0.182	0.088	0.081	0.103	0.126	0.098	0.173	1.292	0.505
NP4	40.691	16.620	1.800	0.306	0.164	0.039	0.044	0.053	0.035	0.107	1.051	0.508
NP5	58.640	28.001	1.836	0.166	0.201	0.052	0.066	0.080	0.038	0.168	1.866	0.586
NP6	37.418	18.855	1.525	0.192	0.104	0.197	0.042	0.043	0.039	0.121	0.689	0.306
NP7	14.172	25.178	0.564	0.157	0.072	0.128	0.058	0.067	0.052	0.085	0.664	0.419
NP8	35.798	24.673	3.358	0.225	0.209	0.104	0.060	0.065	0.053	0.200	1.308	0.723
NP9	49.410	24.260	2.381	0.194	0.121	0.174	0.052	0.060	0.048	0.138	0.948	0.526
NP10	26.820	19.468	0.909	0.137	0.064	0.152	0.047	0.063	0.032	0.070	1.246	0.645
NP11	45.441	29.515	1.599	0.138	0.095	0.050	0.039	0.044	0.050	0.136	0.517	0.243
NP12	51.935	31.329	2.525	0.303	0.185	0.080	0.085	0.099	0.054	0.232	1.258	0.590
NP13	23.198	13.286	1.368	0.187	0.081	0.063	0.141	0.202	0.064	0.060	0.673	0.433
NP14	27.435	15.109	1.799	0.158	0.098	0.039	0.042	0.058	0.035	0.066	0.893	0.450
NP15	17.623	15.084	1.254	0.118	0.066	0.064	0.053	0.064	0.031	0.062	0.716	0.317
NP16	14.046	8.397	0.864	0.164	0.083	0.019	0.045	0.069	0.022	0.034	0.570	0.328
NP17	22.509	14.651	2.044	0.239	0.093	0.056	0.084	0.113	0.041	0.064	1.102	0.652
NP18	23.218	12.537	1.138	0.161	0.079	0.064	0.132	0.189	0.067	0.055	0.725	0.362
NP19	38.828	26.173	1.682	0.157	0.103	0.077	0.081	0.101	0.063	0.157	1.373	0.585
NP20	24.147	13.235	0.883	0.161	0.063	0.055	0.049	0.069	0.043	0.060	0.583	0.354
NP21	28.882	11.950	0.631	0.137	0.181	0.036	0.037	0.042	0.052	0.080	0.604	0.272
NP22	35.504	13.700	0.687	0.149	0.094	0.058	0.059	0.074	0.060	0.087	0.845	0.441
NP23	27.324	17.085	0.940	0.166	0.148	0.093	0.042	0.055	0.066	0.191	0.912	0.381
NP24	19.105	15.990	0.950	0.167	0.111	0.059	0.084	0.123	0.098	0.144	1.369	0.338
NP25	17.379	9.304	0.710	0.160	0.094	0.050	0.069	0.090	0.063	0.080	1.165	0.321
NP26	34.907	13.576	0.553	0.149	0.126	0.157	0.037	0.042	0.040	0.083	0.744	0.375

NP27	23.979	13.758	0.624	0.152	0.090	0.154	0.071	0.084	0.073	0.087	0.460	0.171
NP28	39.953	17.683	1.759	0.270	0.222	0.145	0.072	0.090	0.101	0.135	1.403	0.585
NP29	24.078	10.055	1.587	0.112	0.144	0.107	0.037	0.042	0.031	0.090	0.638	0.285
NP30	26.623	12.644	1.491	0.135	0.114	0.167	0.066	0.079	0.077	0.101	0.760	0.305
NP31	24.487	12.218	1.008	0.147	0.211	0.028	0.026	0.029	0.030	0.057	0.512	0.225
NP32	39.468	25.878	3.402	0.338	0.365	0.077	0.070	0.078	0.050	0.107	0.646	0.279
NP33	27.244	11.506	1.290	0.087	0.084	0.031	0.035	0.048	0.027	0.114	0.594	0.323
NP34	33.356	15.679	0.839	0.130	0.107	0.047	0.048	0.063	0.051	0.133	0.793	0.403
NP35	27.162	5.904	0.530	0.122	0.114	0.024	0.025	0.029	0.041	0.116	0.753	0.347
NP36	33.038	16.125	2.097	0.319	0.179	0.070	0.062	0.077	0.079	0.281	2.066	0.654
NP37	32.913	11.524	0.930	0.256	0.117	0.073	0.052	0.068	0.054	0.103	0.728	0.295
NP38	23.184	15.541	0.647	0.162	0.166	0.068	0.058	0.074	0.065	0.162	1.360	0.597
NP39	21.988	11.304	0.588	0.120	0.065	0.090	0.037	0.051	0.057	0.098	1.207	0.455
NP40	26.433	13.264	2.201	0.160	0.273	0.050	0.048	0.067	0.068	0.127	0.813	0.327
NP41	24.773	22.782	1.669	0.321	0.133	0.074	0.058	0.073	0.069	0.158	3.045	1.047
NP42	26.046	30.093	2.536	0.148	0.091	0.036	0.036	0.042	0.054	0.172	2.929	0.934
NP43	27.329	21.631	1.026	0.127	0.092	0.038	0.036	0.043	0.028	0.094	1.733	0.624
NP44	22.852	25.446	1.833	0.118	0.064	0.040	0.037	0.057	0.071	0.203	2.874	0.846
NP45	26.851	25.645	2.429	0.234	0.188	0.075	0.049	0.052	0.082	0.281	3.634	1.050
NP46	14.372	26.610	1.327	0.196	0.077	0.052	0.067	0.099	0.142	0.284	3.762	1.058
NP47	19.038	36.376	1.720	0.179	0.119	0.063	0.055	0.067	0.085	0.233	3.525	0.777
NP48	28.195	31.604	2.605	0.209	0.166	0.034	0.035	0.047	0.080	0.248	4.088	1.193
NP49	18.598	20.887	2.148	0.190	0.198	0.033	0.057	0.079	0.088	0.185	2.748	0.658
NP50	21.881	25.063	1.877	0.170	0.090	0.076	0.037	0.043	0.063	0.189	3.529	1.045
NP51	17.013	25.820	1.837	0.176	0.087	0.101	0.059	0.087	0.104	0.260	4.080	1.010
NP52	24.265	26.381	2.335	0.254	0.133	0.098	0.041	0.055	0.078	0.242	1.654	0.554

NP53	18.907	15.145	0.929	0.198	0.084	0.129	0.038	0.047	0.067	0.144	1.469	0.633
NP54	19.004	27.144	1.756	0.263	0.122	0.069	0.057	0.083	0.116	0.317	5.407	1.278
NP55	19.116	29.566	1.739	0.215	0.086	0.086	0.055	0.091	0.090	0.211	4.817	1.063
NP56	27.037	22.657	2.624	0.192	0.170	0.050	0.058	0.067	0.046	0.134	2.505	0.802
NP57	19.756	19.207	1.144	0.139	0.102	0.061	0.046	0.049	0.043	0.112	1.887	0.735
NP58	26.024	21.482	1.536	0.276	0.239	0.113	0.104	0.079	0.143	0.171	2.257	0.872
NP59	25.245	29.730	2.166	0.154	0.102	0.044	0.039	0.050	0.043	0.136	2.711	0.676
NP60	28.963	28.064	4.886	0.292	0.209	0.054	0.068	0.081	0.113	0.256	3.229	1.076
NP61	18.998	14.186	1.082	0.166	0.114	0.05	0.084	0.071	0.089	0.132	1.564	0.782
NP62	40.762	27.265	3.371	0.184	0.175	0.057	0.083	0.068	0.117	0.27	1.764	0.892
NP63	28.764	15.565	2.204	0.191	0.149	0.08	0.141	0.09	0.066	0.137	1.599	0.583
NP64	25.599	17.844	3.379	0.206	0.148	0.049	0.049	0.041	0.064	0.136	1.886	1.002
NP65	17.013	13.788	1.185	0.177	0.104	0.043	0.057	0.055	0.082	0.151	1.615	0.603
NP66	22.028	13.418	0.824	0.161	0.169	0.04	0.071	0.045	0.077	0.148	1.5	0.624
NP67	26.403	18.339	1.288	0.177	0.114	0.049	0.09	0.071	0.096	0.185	1.873	0.647
NP68	21.274	16.51	1.742	0.191	0.101	0.058	0.089	0.062	0.071	0.101	1.521	0.631
NP69	21.927	20.017	1.612	0.154	0.081	0.064	0.089	0.074	0.126	0.196	3.366	0.879
NP70	31.136	22.082	3.534	0.209	0.181	0.037	0.09	0.05	0.114	0.23	1.845	0.948
NP71	44.786	32.728	3.134	0.309	0.222	0.082	0.093	0.099	0.182	0.432	3.812	1.403
NP72	22.918	15.549	1.087	0.159	0.148	0.067	0.085	0.078	0.073	0.152	1.499	0.563
NP73	21.224	17.205	1.197	0.177	0.122	0.058	0.086	0.094	0.133	0.142	1.987	0.755
NP74	14.746	17.462	0.955	0.202	0.103	0.057	0.093	0.094	0.192	0.244	2.391	0.651
NP75	38.713	30.926	4.262	0.219	0.18	0.076	0.077	0.064	0.096	0.277	3.022	1.115
NP76	28.148	19.796	1.966	0.302	0.156	0.062	0.089	0.082	0.101	0.179	2.424	0.893
NP77	33.375	25.03	3.047	0.26	0.207	0.07	0.066	0.068	0.105	0.223	3.495	1.19
NP78	29.733	20.613	1.751	0.159	0.132	0.07	0.121	0.137	0.133	0.194	2.797	0.893

NP79	22.997	28.511	1.392	0.235	0.102	0.097	0.174	0.186	0.293	0.342	3.867	1.13
NP80	29.083	17.992	1.572	0.189	0.149	0.054	0.09	0.099	0.159	0.176	1.803	0.781
M_NP	26.418	18.597	1.580	0.177	0.118	0.063	0.058	0.068	0.067	0.143	1.500	0.619
Q1_NP	21.973	14.535	1.006	0.156	0.093	0.049	0.045	0.053	0.050	0.100	0.837	0.398
Q3_NP	32.944	25.496	2.110	0.211	0.167	0.080	0.084	0.085	0.096	0.197	2.557	0.874

C0, carnitine; C2, acetylcarnitine; C3, propionylcarnitine; C4, butyrylcarnitine; C5, isovalerylcarnitine; C6, caproylcarnitine; C8, caprylylcarnitine; C10, actinylcarnitine, C12, lauroylcarnitine; C14, myristoylcarnitine; C16, palmitoylcarnitine; C18, octadecylcarnitine; M, Median; Q, Quartile; P, PCD; NP, NoPCD

Supplementary Table S3. Levels of various acylcarnitines summations in dried blood specimens (μmol/L).

Case ID	C4+C5	C4+C2	C4+C14	C4+C3	C5+C2	C5+C14	C5+C3	C2+C14	C2+C3	C14+C3	C4+C5+C2	C4+C5+C14	C4+C5+C3	C5+C2+C14	C5+C2+C3	C2+C14+C3	C4+C5+C2+C14	C5+C2+C14+C3	C4+C5+C2+C14+C3	C3+C16
P1	0.054	3.009	0.057	0.314	2.983	0.031	0.288	2.986	3.243	0.291	3.023	0.071	0.328	3.000	3.257	3.260	3.040	3.274	3.314	0.462
P2	0.101	5.133	0.075	0.245	5.116	0.058	0.228	5.090	5.260	0.202	5.175	0.117	0.287	5.132	5.302	5.276	5.191	5.318	5.377	0.270
P3	0.059	4.859	0.051	0.238	4.856	0.048	0.235	4.848	5.035	0.227	4.887	0.079	0.266	4.876	5.063	5.055	4.907	5.083	5.114	0.377
P4	0.053	4.979	0.050	0.189	4.990	0.061	0.200	4.987	5.126	0.197	5.011	0.082	0.221	5.019	5.158	5.155	5.040	5.187	5.208	0.378
P5	0.038	2.871	0.039	0.142	2.877	0.045	0.148	2.878	2.981	0.149	2.893	0.061	0.164	2.900	3.003	3.004	2.916	3.026	3.042	0.230
P6	0.085	7.436	0.082	0.611	7.419	0.065	0.594	7.416	7.945	0.591	7.470	0.116	0.645	7.450	7.979	7.976	7.501	8.010	8.061	0.785
P7	0.155	7.555	0.112	0.579	7.576	0.133	0.600	7.533	8.000	0.557	7.643	0.200	0.667	7.621	8.088	8.045	7.688	8.133	8.200	0.765
P8	0.059	3.644	0.052	0.215	3.645	0.053	0.216	3.638	3.801	0.209	3.674	0.082	0.245	3.668	3.831	3.824	3.697	3.854	3.883	0.264
P9	0.081	4.458	0.072	0.199	4.437	0.051	0.178	4.428	4.555	0.169	4.488	0.102	0.229	4.458	4.585	4.576	4.509	4.606	4.657	0.243
P10	0.131	4.501	0.110	0.377	4.448	0.057	0.324	4.427	4.694	0.303	4.540	0.149	0.416	4.466	4.733	4.712	4.558	4.751	4.843	0.377
P11	0.163	7.103	0.098	0.186	7.178	0.173	0.261	7.113	7.201	0.196	7.222	0.217	0.305	7.232	7.320	7.255	7.276	7.374	7.418	0.349
P12	0.067	4.522	0.063	0.150	4.507	0.048	0.135	4.503	4.590	0.131	4.548	0.089	0.176	4.529	4.616	4.612	4.570	4.638	4.679	0.222
P13	0.088	5.917	0.084	0.499	5.891	0.058	0.473	5.887	6.302	0.469	5.948	0.115	0.530	5.918	6.333	6.329	5.975	6.360	6.417	0.639
P14	0.074	6.534	0.086	0.253	6.516	0.068	0.235	6.528	6.695	0.247	6.562	0.114	0.281	6.556	6.723	6.735	6.602	6.763	6.809	0.535
P15	0.103	4.299	0.092	0.484	4.254	0.047	0.439	4.243	4.635	0.428	4.328	0.121	0.513	4.272	4.664	4.653	4.346	4.682	4.756	0.692
P16	0.058	2.862	0.055	0.150	2.844	0.037	0.132	2.841	2.936	0.129	2.882	0.075	0.170	2.861	2.956	2.953	2.899	2.973	3.011	0.190
P17	0.096	7.412	0.074	0.301	7.426	0.088	0.315	7.404	7.631	0.293	7.467	0.129	0.356	7.459	7.686	7.664	7.500	7.719	7.760	0.498
P18	0.073	4.382	0.061	0.150	4.363	0.042	0.131	4.351	4.440	0.119	4.409	0.088	0.177	4.378	4.467	4.455	4.424	4.482	4.528	0.337
P19	0.100	7.247	0.075	0.367	7.239	0.067	0.359	7.214	7.506	0.334	7.293	0.121	0.413	7.260	7.552	7.527	7.314	7.573	7.627	0.556
P20	0.025	1.086	0.038	0.063	1.077	0.029	0.054	1.090	1.115	0.067	1.094	0.046	0.071	1.098	1.123	1.136	1.115	1.144	1.161	0.088
P21	0.020	0.700	0.031	0.059	0.692	0.023	0.051	0.703	0.731	0.062	0.706	0.037	0.065	0.709	0.737	0.748	0.723	0.754	0.768	0.106
P22	0.066	4.311	0.068	0.370	4.299	0.056	0.358	4.301	4.603	0.360	4.338	0.095	0.397	4.328	4.630	4.632	4.367	4.659	4.698	0.484

P23	0.080	2.740	0.070	0.260	2.720	0.050	0.240	2.710	2.900	0.230	2.770	0.100	0.290	2.740	2.930	2.920	2.790	2.950	3.000	0.380
P24	0.120	9.790	0.140	0.900	9.770	0.120	0.880	9.790	10.550	0.900	9.840	0.190	0.950	9.840	10.600	10.620	9.910	10.670	10.740	1.040
P25	0.110	3.170	0.090	0.310	3.160	0.080	0.300	3.140	3.360	0.280	3.220	0.140	0.360	3.190	3.410	3.390	3.250	3.440	3.500	0.610
P26	0.100	3.190	0.100	0.360	3.150	0.060	0.320	3.150	3.410	0.320	3.220	0.130	0.390	3.180	3.440	3.440	3.250	3.470	3.540	0.580
P27	0.080	2.970	0.080	0.310	2.950	0.060	0.290	2.950	3.180	0.290	3.000	0.110	0.340	2.980	3.210	3.210	3.030	3.240	3.290	0.580
P28	0.071	4.324	0.075	0.175	4.308	0.059	0.159	4.311	4.411	0.162	4.351	0.102	0.203	4.339	4.439	4.442	4.382	4.470	4.514	0.367
P29	0.072	3.444	0.073	0.422	3.418	0.047	0.397	3.419	3.769	0.397	3.467	0.096	0.445	3.442	3.792	3.793	3.491	3.816	3.865	0.626
P30	0.125	6.417	0.115	1.501	6.391	0.090	1.476	6.382	7.768	1.467	6.466	0.165	1.551	6.432	7.818	7.808	6.507	7.858	7.933	1.631
P31	0.080	5.840	0.100	0.370	5.800	0.060	0.330	5.820	6.090	0.350	5.860	0.120	0.390	5.840	6.110	6.130	5.900	6.150	6.210	0.820
P32	0.112	4.872	0.085	0.443	4.856	0.069	0.427	4.830	5.187	0.400	4.920	0.133	0.491	4.878	5.235	5.209	4.942	5.257	5.320	0.559
P33	0.100	9.360	0.140	0.700	9.340	0.120	0.680	9.380	9.940	0.720	9.400	0.180	0.740	9.420	9.980	10.020	9.480	10.060	10.120	1.460
P34	0.124	4.302	0.092	0.455	4.291	0.081	0.445	4.259	4.622	0.412	4.358	0.149	0.512	4.315	4.679	4.647	4.383	4.704	4.771	0.571
P35	0.170	9.070	0.200	1.120	9.060	0.190	1.110	9.090	10.010	1.140	9.150	0.280	1.200	9.170	10.090	10.120	9.260	10.200	10.290	2.060
P36	0.073	2.788	0.067	0.322	2.787	0.066	0.320	2.781	3.035	0.315	2.824	0.103	0.357	2.817	3.071	3.065	2.854	3.101	3.138	0.523
P37	0.110	9.570	0.140	0.950	9.540	0.110	0.920	9.570	10.380	0.950	9.610	0.180	0.990	9.610	10.420	10.450	9.680	10.490	10.560	2.020
P38	0.073	5.115	0.092	0.363	5.118	0.095	0.366	5.137	5.408	0.385	5.153	0.130	0.401	5.175	5.446	5.465	5.210	5.503	5.538	0.785
P39	0.140	10.270	0.130	0.860	10.250	0.110	0.840	10.240	10.970	0.830	10.330	0.190	0.920	10.300	11.030	11.020	10.380	11.080	11.160	1.460
P40	0.124	4.069	0.103	0.406	4.069	0.103	0.406	4.048	4.351	0.385	4.131	0.165	0.468	4.110	4.413	4.392	4.172	4.454	4.516	0.566
P41	0.080	6.110	0.100	0.490	6.090	0.080	0.470	6.110	6.500	0.490	6.140	0.130	0.520	6.140	6.530	6.550	6.190	6.580	6.630	0.970
P42	0.054	3.905	0.043	0.094	3.913	0.051	0.102	3.902	3.953	0.091	3.936	0.074	0.125	3.933	3.984	3.973	3.956	4.004	4.027	0.149
P43	0.103	10.716	0.137	0.645	10.669	0.090	0.598	10.703	11.211	0.632	10.744	0.165	0.673	10.731	11.239	11.273	10.806	11.301	11.376	1.677
P44	0.085	7.436	0.082	0.611	7.419	0.065	0.594	7.416	7.945	0.591	7.470	0.116	0.645	7.450	7.979	7.976	7.501	8.010	8.061	0.785
P45	0.060	2.540	0.080	0.520	2.520	0.060	0.500	2.540	2.980	0.520	2.560	0.100	0.540	2.560	3.000	3.020	2.600	3.040	3.080	1.000
P46	0.155	7.555	0.112	0.579	7.576	0.133	0.600	7.533	8.000	0.557	7.643	0.200	0.667	7.621	8.088	8.045	7.688	8.133	8.200	0.765
P47	0.128	12.199	0.185	0.886	12.159	0.145	0.846	12.216	12.917	0.903	12.243	0.229	0.930	12.260	12.961	13.018	12.344	13.062	13.146	2.060
P48	0.120	9.638	0.147	0.721	9.598	0.107	0.681	9.625	10.199	0.708	9.678	0.187	0.761	9.665	10.239	10.266	9.745	10.306	10.386	1.188

P49	0.096	7.412	0.074	0.301	7.426	0.088	0.315	7.404	7.631	0.293	7.467	0.129	0.356	7.459	7.686	7.664	7.500	7.719	7.760	0.498
P50	0.137	10.671	0.186	0.871	10.628	0.143	0.828	10.677	11.362	0.877	10.718	0.233	0.918	10.724	11.409	11.458	10.814	11.505	11.595	1.692
P51	0.090	3.160	0.081	0.277	3.132	0.053	0.249	3.123	3.319	0.240	3.191	0.112	0.308	3.154	3.350	3.341	3.213	3.372	3.431	0.333
P52	0.126	9.625	0.188	0.918	9.581	0.144	0.874	9.643	10.373	0.936	9.666	0.229	0.959	9.684	10.414	10.476	9.769	10.517	10.602	2.453
P53	0.095	2.864	0.098	0.246	2.841	0.075	0.223	2.844	2.992	0.226	2.900	0.134	0.282	2.880	3.028	3.031	2.939	3.067	3.126	0.316
P54	0.161	6.013	0.166	0.382	5.966	0.119	0.335	5.971	6.187	0.340	6.070	0.223	0.439	6.028	6.244	6.249	6.132	6.306	6.410	0.798
P55	0.116	9.670	0.129	0.575	9.644	0.103	0.549	9.657	10.103	0.562	9.715	0.174	0.620	9.702	10.148	10.161	9.773	10.206	10.277	0.998
P56	0.164	7.761	0.180	0.982	7.707	0.126	0.928	7.723	8.525	0.944	7.816	0.235	1.037	7.778	8.580	8.596	7.887	8.651	8.760	1.867
P57	0.120	6.410	0.110	0.470	6.390	0.090	0.450	6.380	6.740	0.440	6.460	0.160	0.520	6.430	6.790	6.780	6.500	6.830	6.900	1.290
P58	0.092	6.709	0.083	0.392	6.695	0.069	0.378	6.686	6.995	0.369	6.748	0.122	0.431	6.725	7.034	7.025	6.778	7.064	7.117	0.806
P59	0.133	7.321	0.142	0.505	7.298	0.119	0.482	7.307	7.670	0.491	7.376	0.197	0.560	7.362	7.725	7.734	7.440	7.789	7.867	1.389
P60	0.118	7.801	0.111	0.427	7.803	0.113	0.429	7.796	8.112	0.422	7.861	0.171	0.487	7.856	8.172	8.165	7.914	8.225	8.283	1.067
P61	0.142	7.410	0.151	0.630	7.344	0.085	0.564	7.353	7.832	0.573	7.448	0.189	0.668	7.391	7.870	7.879	7.495	7.917	8.021	1.108
P62	0.085	7.585	0.098	0.428	7.580	0.093	0.423	7.593	7.923	0.436	7.625	0.138	0.468	7.633	7.963	7.976	7.678	8.016	8.061	1.111
P63	0.131	7.204	0.139	0.332	7.167	0.102	0.295	7.175	7.367	0.302	7.251	0.186	0.379	7.222	7.415	7.422	7.306	7.469	7.553	0.627
P64	0.181	10.231	0.197	0.739	10.159	0.125	0.666	10.175	10.717	0.683	10.285	0.251	0.793	10.229	10.771	10.787	10.356	10.841	10.968	1.543
P65	0.058	7.191	0.080	0.269	7.182	0.072	0.260	7.205	7.393	0.283	7.215	0.105	0.293	7.229	7.418	7.441	7.262	7.465	7.498	0.514
P66	0.080	6.444	0.105	0.445	6.437	0.098	0.438	6.461	6.802	0.463	6.480	0.141	0.482	6.498	6.838	6.863	6.542	6.899	6.943	1.338
P67	0.114	9.467	0.133	0.502	9.449	0.115	0.484	9.468	9.837	0.503	9.515	0.181	0.550	9.516	9.885	9.904	9.582	9.952	10.018	1.357
P68	0.064	7.284	0.102	0.451	7.289	0.106	0.456	7.326	7.676	0.493	7.318	0.136	0.485	7.361	7.710	7.748	7.390	7.782	7.812	1.487
P69	0.043	2.605	0.082	0.293	2.596	0.073	0.285	2.635	2.847	0.324	2.622	0.098	0.310	2.652	2.864	2.903	2.678	2.920	2.946	0.643
P70	0.109	9.490	0.164	0.706	9.455	0.129	0.671	9.510	10.052	0.726	9.527	0.201	0.743	9.547	10.089	10.144	9.619	10.181	10.253	1.825
P71	0.104	6.192	0.115	0.589	6.168	0.091	0.565	6.179	6.653	0.576	6.232	0.155	0.629	6.219	6.693	6.704	6.283	6.744	6.808	1.450
P72	0.121	7.172	0.152	1.285	7.135	0.115	1.248	7.166	8.299	1.279	7.214	0.194	1.327	7.208	8.341	8.372	7.287	8.414	8.493	2.104
M_P	0.098	6.301	0.095	0.414	6.279	0.080	0.401	6.280	6.674	0.391	6.346	0.132	0.457	6.325	6.708	6.720	6.392	6.754	6.809	0.668
Q1_P	0.073	4.242	0.074	0.275	4.208	0.058	0.261	4.194	4.396	0.272	4.279	0.103	0.307	4.232	4.432	4.430	4.303	4.466	4.515	0.442

Q3_P	0.122	7.555	0.131	0.595	7.576	0.110	0.594	7.533	8.000	0.574	7.630	0.182	0.651	7.621	8.088	8.045	7.681	8.133	8.200	1.302
NP1	0.310	19.232	0.249	1.581	19.224	0.241	1.573	19.163	20.495	1.512	19.383	0.400	1.732	19.314	20.646	20.585	19.473	20.736	20.895	2.339
NP2	0.314	16.898	0.250	1.337	16.834	0.186	1.273	16.770	17.857	1.209	17.023	0.375	1.462	16.895	17.982	17.918	17.084	18.043	18.232	2.238
NP3	0.270	23.499	0.355	1.180	23.405	0.261	1.086	23.490	24.315	1.171	23.587	0.443	1.268	23.578	24.403	24.488	23.760	24.576	24.758	2.290
NP4	0.470	16.926	0.413	2.106	16.784	0.271	1.964	16.727	18.420	1.907	17.090	0.577	2.270	16.891	18.584	18.527	17.197	18.691	18.997	2.851
NP5	0.367	28.167	0.334	2.002	28.202	0.369	2.037	28.169	29.837	2.004	28.368	0.535	2.203	28.370	30.038	30.005	28.536	30.206	30.372	3.702
NP6	0.296	19.047	0.313	1.717	18.959	0.225	1.629	18.976	20.380	1.646	19.151	0.417	1.821	19.080	20.484	20.501	19.272	20.605	20.797	2.214
NP7	0.229	25.335	0.242	0.721	25.250	0.157	0.636	25.263	25.742	0.649	25.407	0.314	0.793	25.335	25.814	25.827	25.492	25.899	26.056	1.228
NP8	0.434	24.898	0.425	3.583	24.882	0.409	3.567	24.873	28.031	3.558	25.107	0.634	3.792	25.082	28.240	28.231	25.307	28.440	28.665	4.666
NP9	0.315	24.454	0.332	2.575	24.381	0.259	2.502	24.398	26.641	2.519	24.575	0.453	2.696	24.519	26.762	26.779	24.713	26.900	27.094	3.329
NP10	0.201	19.605	0.207	1.046	19.532	0.134	0.973	19.538	20.377	0.979	19.669	0.271	1.110	19.602	20.441	20.447	19.739	20.511	20.648	2.155
NP11	0.233	29.653	0.274	1.737	29.610	0.231	1.694	29.651	31.114	1.735	29.748	0.369	1.832	29.746	31.209	31.250	29.884	31.345	31.483	2.116
NP12	0.488	31.632	0.535	2.828	31.514	0.417	2.710	31.561	33.854	2.757	31.817	0.720	3.013	31.746	34.039	34.086	32.049	34.271	34.574	3.783
NP13	0.268	13.473	0.247	1.555	13.367	0.141	1.449	13.346	14.654	1.428	13.554	0.328	1.636	13.427	14.735	14.714	13.614	14.795	14.982	2.041
NP14	0.256	15.267	0.224	1.957	15.207	0.164	1.897	15.175	16.908	1.865	15.365	0.322	2.055	15.273	17.006	16.974	15.431	17.072	17.230	2.692
NP15	0.184	15.202	0.180	1.372	15.150	0.128	1.320	15.146	16.338	1.316	15.268	0.246	1.438	15.212	16.404	16.400	15.330	16.466	16.584	1.970
NP16	0.247	8.561	0.198	1.028	8.480	0.117	0.947	8.431	9.261	0.898	8.644	0.281	1.111	8.514	9.344	9.295	8.678	9.378	9.542	1.434
NP17	0.332	14.890	0.303	2.283	14.744	0.157	2.137	14.715	16.695	2.108	14.983	0.396	2.376	14.808	16.788	16.759	15.047	16.852	17.091	3.146
NP18	0.240	12.698	0.216	1.299	12.616	0.134	1.217	12.592	13.675	1.193	12.777	0.295	1.378	12.671	13.754	13.730	12.832	13.809	13.970	1.863
NP19	0.260	26.330	0.314	1.839	26.276	0.260	1.785	26.330	27.855	1.839	26.433	0.417	1.942	26.433	27.958	28.012	26.590	28.115	28.272	3.055
NP20	0.224	13.396	0.221	1.044	13.298	0.123	0.946	13.295	14.118	0.943	13.459	0.284	1.107	13.358	14.181	14.178	13.519	14.241	14.402	1.466
NP21	0.318	12.087	0.217	0.768	12.131	0.261	0.812	12.030	12.581	0.711	12.268	0.398	0.949	12.211	12.762	12.661	12.348	12.842	12.979	1.235
NP22	0.243	13.849	0.236	0.836	13.794	0.181	0.781	13.787	14.387	0.774	13.943	0.330	0.930	13.881	14.481	14.474	14.030	14.568	14.717	1.532
NP23	0.314	17.251	0.357	1.106	17.233	0.339	1.088	17.276	18.025	1.131	17.399	0.505	1.254	17.424	18.173	18.216	17.590	18.364	18.530	1.852
NP24	0.278	16.157	0.311	1.117	16.101	0.255	1.061	16.134	16.940	1.094	16.268	0.422	1.228	16.245	17.051	17.084	16.412	17.195	17.362	2.319
NP25	0.254	9.464	0.240	0.870	9.398	0.174	0.804	9.384	10.014	0.790	9.558	0.334	0.964	9.478	10.108	10.094	9.638	10.188	10.348	1.875

NP26	0.275	13.725	0.232	0.702	13.702	0.209	0.679	13.659	14.129	0.636	13.851	0.358	0.828	13.785	14.255	14.212	13.934	14.338	14.487	1.297
NP27	0.242	13.910	0.239	0.776	13.848	0.177	0.714	13.845	14.382	0.711	14.000	0.329	0.866	13.935	14.472	14.469	14.087	14.559	14.711	1.084
NP28	0.492	17.953	0.405	2.029	17.905	0.357	1.981	17.818	19.442	1.894	18.175	0.627	2.251	18.040	19.664	19.577	18.310	19.799	20.069	3.162
NP29	0.256	10.167	0.202	1.699	10.199	0.234	1.731	10.145	11.642	1.677	10.311	0.346	1.843	10.289	11.786	11.732	10.401	11.876	11.988	2.225
NP30	0.249	12.779	0.236	1.626	12.758	0.215	1.605	12.745	14.135	1.592	12.893	0.350	1.740	12.859	14.249	14.236	12.994	14.350	14.485	2.251
NP31	0.358	12.365	0.204	1.155	12.429	0.268	1.219	12.275	13.226	1.065	12.576	0.415	1.366	12.486	13.437	13.283	12.633	13.494	13.641	1.520
NP32	0.703	26.216	0.445	3.740	26.243	0.472	3.767	25.985	29.280	3.509	26.581	0.810	4.105	26.350	29.645	29.387	26.688	29.752	30.090	4.048
NP33	0.171	11.593	0.201	1.377	11.590	0.198	1.374	11.620	12.796	1.404	11.677	0.285	1.461	11.704	12.880	12.910	11.791	12.994	13.081	1.884
NP34	0.237	15.809	0.263	0.969	15.786	0.240	0.946	15.812	16.518	0.972	15.916	0.370	1.076	15.919	16.625	16.651	16.049	16.758	16.888	1.632
NP35	0.236	6.026	0.238	0.652	6.018	0.230	0.644	6.020	6.434	0.646	6.140	0.352	0.766	6.134	6.548	6.550	6.256	6.664	6.786	1.283
NP36	0.498	16.444	0.600	2.416	16.304	0.460	2.276	16.406	18.222	2.378	16.623	0.779	2.595	16.585	18.401	18.503	16.904	18.682	19.001	4.163
NP37	0.373	11.780	0.359	1.186	11.641	0.220	1.047	11.627	12.454	1.033	11.897	0.476	1.303	11.744	12.571	12.557	12.000	12.674	12.930	1.658
NP38	0.328	15.703	0.324	0.809	15.707	0.328	0.813	15.703	16.188	0.809	15.869	0.490	0.975	15.869	16.354	16.350	16.031	16.516	16.678	2.007
NP39	0.185	11.424	0.218	0.708	11.369	0.163	0.653	11.402	11.892	0.686	11.489	0.283	0.773	11.467	11.957	11.990	11.587	12.055	12.175	1.795
NP40	0.433	13.424	0.287	2.361	13.537	0.400	2.474	13.391	15.465	2.328	13.697	0.560	2.634	13.664	15.738	15.592	13.824	15.865	16.025	3.014
NP41	0.454	23.103	0.479	1.990	22.915	0.291	1.802	22.940	24.451	1.827	23.236	0.612	2.123	23.073	24.584	24.609	23.394	24.742	25.063	4.714
NP42	0.239	30.241	0.320	2.684	30.184	0.263	2.627	30.265	32.629	2.708	30.332	0.411	2.775	30.356	32.720	32.801	30.504	32.892	33.040	5.465
NP43	0.219	21.758	0.221	1.153	21.723	0.186	1.118	21.725	22.657	1.120	21.850	0.313	1.245	21.817	22.749	22.751	21.944	22.843	22.970	2.759
NP44	0.182	25.564	0.321	1.951	25.510	0.267	1.897	25.649	27.279	2.036	25.628	0.385	2.015	25.713	27.343	27.482	25.831	27.546	27.664	4.707
NP45	0.422	25.879	0.515	2.663	25.833	0.469	2.617	25.926	28.074	2.710	26.067	0.703	2.851	26.114	28.262	28.355	26.348	28.543	28.777	6.063
NP46	0.273	26.806	0.480	1.523	26.687	0.361	1.404	26.894	27.937	1.611	26.883	0.557	1.600	26.971	28.014	28.221	27.167	28.298	28.494	5.089
NP47	0.298	36.555	0.412	1.899	36.495	0.352	1.839	36.609	38.096	1.953	36.674	0.531	2.018	36.728	38.215	38.329	36.907	38.448	38.627	5.245
NP48	0.375	31.813	0.457	2.814	31.770	0.414	2.771	31.852	34.209	2.853	31.979	0.623	2.980	32.018	34.375	34.457	32.227	34.623	34.832	6.693
NP49	0.388	21.077	0.375	2.338	21.085	0.383	2.346	21.072	23.035	2.333	21.275	0.573	2.536	21.270	23.233	23.220	21.460	23.418	23.608	4.896
NP50	0.260	25.233	0.359	2.047	25.153	0.279	1.967	25.252	26.940	2.066	25.323	0.449	2.137	25.342	27.030	27.129	25.512	27.219	27.389	5.406
NP51	0.263	25.996	0.436	2.013	25.907	0.347	1.924	26.080	27.657	2.097	26.083	0.523	2.100	26.167	27.744	27.917	26.343	28.004	28.180	5.917

NP52	0.387	26.635	0.496	2.589	26.514	0.375	2.468	26.623	28.716	2.577	26.768	0.629	2.722	26.756	28.849	28.958	27.010	29.091	29.345	3.989
NP53	0.282	15.343	0.342	1.127	15.229	0.228	1.013	15.289	16.074	1.073	15.427	0.426	1.211	15.373	16.158	16.218	15.571	16.302	16.500	2.398
NP54	0.385	27.407	0.580	2.019	27.266	0.439	1.878	27.461	28.900	2.073	27.529	0.702	2.141	27.583	29.022	29.217	27.846	29.339	29.602	7.163
NP55	0.301	29.781	0.426	1.954	29.652	0.297	1.825	29.777	31.305	1.950	29.867	0.512	2.040	29.863	31.391	31.516	30.078	31.602	31.817	6.556
NP56	0.362	22.849	0.326	2.816	22.827	0.304	2.794	22.791	25.281	2.758	23.019	0.496	2.986	22.961	25.451	25.415	23.153	25.585	25.777	5.129
NP57	0.241	19.346	0.251	1.283	19.309	0.214	1.246	19.319	20.351	1.256	19.448	0.353	1.385	19.421	20.453	20.463	19.560	20.565	20.704	3.031
NP58	0.515	21.758	0.447	1.812	21.721	0.410	1.775	21.653	23.018	1.707	21.997	0.686	2.051	21.892	23.257	23.189	22.168	23.428	23.704	3.793
NP59	0.256	29.884	0.290	2.320	29.832	0.238	2.268	29.866	31.896	2.302	29.986	0.392	2.422	29.968	31.998	32.032	30.122	32.134	32.288	4.877
NP60	0.501	28.356	0.548	5.178	28.273	0.465	5.095	28.320	32.950	5.142	28.565	0.757	5.387	28.529	33.159	33.206	28.821	33.415	33.707	8.115
NP61	0.280	14.352	0.298	1.248	14.300	0.246	1.196	14.318	15.268	1.214	14.466	0.412	1.362	14.432	15.382	15.400	14.598	15.514	15.680	2.646
NP62	0.359	27.449	0.454	3.555	27.440	0.445	3.546	27.535	30.636	3.641	27.624	0.629	3.730	27.710	30.811	30.906	27.894	31.081	31.265	5.135
NP63	0.340	15.756	0.328	2.395	15.714	0.286	2.353	15.702	17.769	2.341	15.905	0.477	2.544	15.851	17.918	17.906	16.042	18.055	18.246	3.803
NP64	0.354	18.050	0.342	3.585	17.992	0.284	3.527	17.980	21.223	3.515	18.198	0.490	3.733	18.128	21.371	21.359	18.334	21.507	21.713	5.265
NP65	0.281	13.965	0.328	1.362	13.892	0.255	1.289	13.939	14.973	1.336	14.069	0.432	1.466	14.043	15.077	15.124	14.220	15.228	15.405	2.800
NP66	0.330	13.579	0.309	0.985	13.587	0.317	0.993	13.566	14.242	0.972	13.748	0.478	1.154	13.735	14.411	14.390	13.896	14.559	14.720	2.324
NP67	0.291	18.516	0.362	1.465	18.453	0.299	1.402	18.524	19.627	1.473	18.630	0.476	1.579	18.638	19.741	19.812	18.815	19.926	20.103	3.161
NP68	0.292	16.701	0.292	1.933	16.611	0.202	1.843	16.611	18.252	1.843	16.802	0.393	2.034	16.712	18.353	18.353	16.903	18.454	18.645	3.263
NP69	0.235	20.171	0.350	1.766	20.098	0.277	1.693	20.213	21.629	1.808	20.252	0.431	1.847	20.294	21.710	21.825	20.448	21.906	22.060	4.978
NP70	0.390	22.291	0.439	3.743	22.263	0.411	3.715	22.312	25.616	3.764	22.472	0.620	3.924	22.493	25.797	25.846	22.702	26.027	26.236	5.379
NP71	0.531	33.037	0.741	3.443	32.950	0.654	3.356	33.160	35.862	3.566	33.259	0.963	3.665	33.382	36.084	36.294	33.691	36.516	36.825	6.946
NP72	0.307	15.708	0.311	1.246	15.697	0.300	1.235	15.701	16.636	1.239	15.856	0.459	1.394	15.849	16.784	16.788	16.008	16.936	17.095	2.586
NP73	0.299	17.382	0.319	1.374	17.327	0.264	1.319	17.347	18.402	1.339	17.504	0.441	1.496	17.469	18.524	18.544	17.646	18.666	18.843	3.184
NP74	0.305	17.664	0.446	1.157	17.565	0.347	1.058	17.706	18.417	1.199	17.767	0.549	1.260	17.809	18.520	18.661	18.011	18.764	18.966	3.346
NP75	0.399	31.145	0.496	4.481	31.106	0.457	4.442	31.203	35.188	4.539	31.325	0.676	4.661	31.383	35.368	35.465	31.602	35.645	35.864	7.284
NP76	0.458	20.098	0.481	2.268	19.952	0.335	2.122	19.975	21.762	2.145	20.254	0.637	2.424	20.131	21.918	21.941	20.433	22.097	22.399	4.390
NP77	0.467	25.290	0.483	3.307	25.237	0.430	3.254	25.253	28.077	3.270	25.497	0.690	3.514	25.460	28.284	28.300	25.720	28.507	28.767	6.542

NP78	0.291	20.772	0.353	1.910	20.745	0.326	1.883	20.807	22.364	1.945	20.904	0.485	2.042	20.939	22.496	22.558	21.098	22.690	22.849	4.548
NP79	0.337	28.746	0.577	1.627	28.613	0.444	1.494	28.853	29.903	1.734	28.848	0.679	1.729	28.955	30.005	30.245	29.190	30.347	30.582	5.259
NP80	0.338	18.181	0.365	1.761	18.141	0.325	1.721	18.168	19.564	1.748	18.330	0.514	1.910	18.317	19.713	19.740	18.506	19.889	20.078	3.375
M_NP	0.300	18.782	0.327	1.749	18.706	0.270	1.708	18.750	20.364	1.735	18.891	0.451	1.845	18.859	20.447	20.455	19.044	20.538	20.676	3.154
Q1_NP	0.256	14.756	0.249	1.174	14.633	0.219	1.111	14.616	15.922	1.161	14.854	0.370	1.294	14.714	16.053	16.062	14.935	16.193	16.381	2.145
Q3_NP	0.374	25.643	0.429	2.325	25.591	0.358	2.270	25.718	27.876	2.309	25.738	0.574	2.452	25.813	27.972	28.064	25.959	28.161	28.328	4.882

C2, acetylcarnitine; C3, propionylcarnitine; C4, butyrylcarnitine; C5, isovalerylcarnitine; C14, myristoylcarnitine; C16, palmitoylcarnitine; M, Median; Q, Quartile; P, PCD; NP, NoPCD
